# Deep learning autofluorescence-harmonic microscopy

**DOI:** 10.1038/s41377-022-00768-x

**Published:** 2022-03-29

**Authors:** Binglin Shen, Shaowen Liu, Yanping Li, Ying Pan, Yuan Lu, Rui Hu, Junle Qu, Liwei Liu

**Affiliations:** 1 Key Laboratory of Optoelectronic Devices and Systems of Guangdong Province and Ministry of Education, College of Physics and Optoelectronic Engineering, Shenzhen University, 518060 Shenzhen, China; 2 Shenzhen Meitu Innovation Technology LTD, 518060 Shenzhen, China; 3 China–Japan Union Hospital of Jilin University, 130033 Changchun, China; 4 The Sixth People’s Hospital of Shenzhen, 518052 Shenzhen, China

**Keywords:** Multiphoton microscopy, Biophotonics

## Abstract

Laser scanning microscopy has inherent tradeoffs between imaging speed, field of view (FOV), and spatial resolution due to the limitations of sophisticated mechanical and optical setups, and deep learning networks have emerged to overcome these limitations without changing the system. Here, we demonstrate deep learning autofluorescence-harmonic microscopy (DLAM) based on self-alignment attention-guided residual-in-residual dense generative adversarial networks to close the gap between speed, FOV, and quality. Using the framework, we demonstrate label-free large-field multimodal imaging of clinicopathological tissues with enhanced spatial resolution and running time advantages. Statistical quality assessments show that the attention-guided residual dense connections minimize the persistent noise, distortions, and scanning fringes that degrade the autofluorescence-harmonic images and avoid reconstruction artifacts in the output images. With the advantages of high contrast, high fidelity, and high speed in image reconstruction, DLAM can act as a powerful tool for the noninvasive evaluation of diseases, neural activity, and embryogenesis.

## Abstract (web summary)

A self-alignment attention-guided residual-in-residual dense generative adversarial networks architecture closes the gap between speed, field of view, and image quality of label-free nonlinear optical microscopes which are notoriously slow.

## Graphical abstract

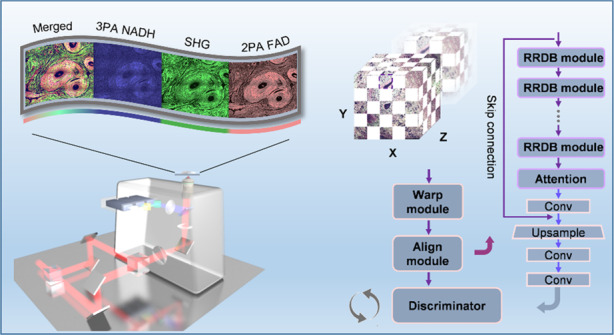

## Introduction

Label-free nonlinear optical microscopy (NLOM)^1–4^, featuring high resolution, deep penetration, low photobleaching, and nonperturbance, can provide abundant structural and functional information and enable a comprehensive and informative analysis of various biochemical phenomena^4–6^. Nonetheless, a confined field of view (FOV) of <600 μm^7^ for a conventional NLOM can hardly realize visualization of large-scale cellular distribution and interactions and possibly lead to incorrect judgment. Large-area investigation of tumor, brain, or other tissues and organs with cellular resolution is a current challenge for NLOM. Typical instrument approaches for expanding the FOV of an NLOM enlarge the diameter of the objective lens^8–10^ or increase the number of objectives^7^, with specifically designed scanning paths. Some reported computational methods, such as structured-illumination microscopy^11^, produce a resolution-enhanced stitched image in the Fourier domain^12^. All these methods require additional costly devices and intricate optical path design. State-of-the-art microscopes equipped with a high-speed sophisticated mechanical scanner (e.g., 720 fps at 2048 × 16 pixels by Nikon AX R) can expediently perform large-area imaging by scanning a series of adjacent FOVs in a short time. However, the resulting images are susceptible to adverse effects, such as background noise, inadequate resolution, and scanning artifacts, which are nonnegligible for label-free nonlinear imaging.

Due to the increasing computing power and quantity of available data, a variety of deep learning methods, ranging from early convolutional neural networks (CNNs) to recent promising generative adversarial networks (GANs), have been proposed and have shown great accomplishments in biomedical imaging^13–20^. This significant progress includes super-resolution^13,15,16^, medical diagnosis^21^, cellular component classification^17^, and virtual H&E staining^14,22^. Among them, super-resolution reconstruction is one of the most important classes of image processing techniques owing to its ability to overcome the limitations of traditional microscopes without changing the system. Deep learning-enhanced super-resolution models can extract morphological details from inferior raw images and attain remarkable resolution improvements for bright-field^13^, fluorescence^15,16,23^, and light-field^18^ microscopy. However, low-resolution images are usually captured using a low-magnification objective lens^12,13^, of which the focusing capability and resulting photon density are insufficient for label-free nonlinear imaging, whereas a direct increase in laser intensity will probably cause photobleaching and photodamage. Additionally, in most cases, the degraded images are generated from the measured high-contrast images with synthesized Gaussian, Poisson, or other noises^12,16,24^. Such computational degradation does not guarantee authenticity because the real situation always has full statistical complexity^25^. Hence, there are increasing demands to develop imaging methods to collect authentic data of both contrary qualities, especially the low-grade domain, to construct a reliable paired training dataset. One feasible approach is to implement fast resonant scanning, which has been extensively applied in two-photon excitation microscopes where wide-field excitation is unavailable. However, demonstrations of efficient, realistic super-resolution models to enhance the performance of fast-scanning NLOM to compete with long-pixel exposure NLOM have not yet been realized.

To obtain large-scale multidimensional information without perturbance while guaranteeing high speed and resolution, we demonstrate deep learning autofluorescence-harmonic microscopy (DLAM) based on the attention-guided residual-in-residual dense generative adversarial network architecture. The network was trained using the preregistered collected dataset, where the trichromatic channels of the images were formed by three typical nonlinear optical processes, including two-photon autofluorescence (2PA) of endogenous flavin adenine dinucleotide (FAD), second-harmonic generation (SHG), and three-photon autofluorescence (3PA) of endogenous nicotinamide adenine dinucleotide (NADH). A label-free multimodal image of human pathological tissues over a 5.4 × 5.4 mm^2^ area at 2176 × 2176 pixels, which was obtained in 54 s using resonant scanning, was transformed into a high-resolution image at 8704 × 8704 pixels within 23 s. For comparison, the time to acquire the same quality image using galvanometer scanning exceeded 10 m. The undesirable noise and scanning artifacts, which were more serious in the 3PA NADH channel, were significantly suppressed, while the semantic information for pathological analyses was fully retained after the deep learning inference. The statistically quantified optical resolution and quality metrics for DLAM exhibited a remarkable increase benefiting from the residual dense connections for the generator with high-level perceptual loss^26^ and a discriminator with spectral normalizations^27,28^. Moreover, DLAM prevented the reconstruction artifacts and avoided the image anamorphoses raised by the conventional GAN models to realize high-authenticity superior-resolution nonlinear imaging.

## Results

### Principle of DLAM

**Fig. 1:**
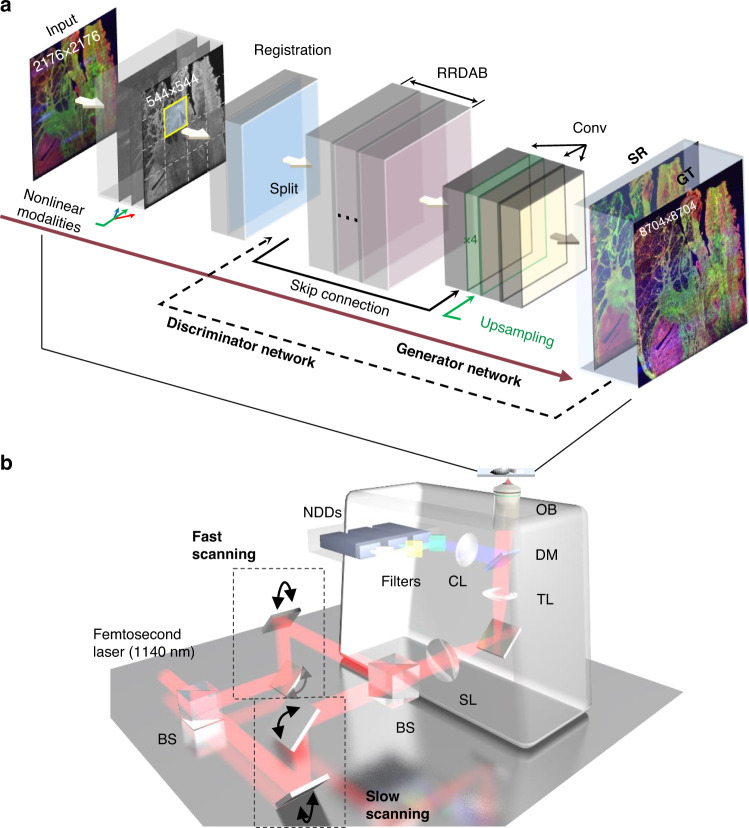
DLAM schematic. **a** The basic network architecture including registration, RRDAB modules, conv layers, the skip connection, upsampling operation, and discriminator. The full frameworks can be found in the section “Materials and methods”, Notes S1–4, and Figs. S1–4. **b** A commercial two-photon microscope with a fast galvo-resonant scanning system and a slow dual-axis galvo scanning system. BS beam splitter, CL collect lens, DM dichroic mirror, NDD nondescanned detectors, OB objective, SL scan lens, TL tube lens.

DLAM combined the proposed deep learning model (Fig. 1a) and the commercial nonlinear optical imaging system (A1R MP+, Nikon), which houses an 8-kHz galvo-resonant (GR) scanning system and a dual-axis galvo (DG) scanning system (Fig. 1b). Two beam splitters (BSs) with motorized shutters switched the excitation light between the two scanning systems. The input images were obtained by the fast resonant scanning mode operating at 30 fps with a frame time of 33.3 ms for 256 × 256 pixels. The target high-quality images approximating the ground truth (GT), were taken by the slow DG scanning system at 0.474 fps with a frame time of 2.1 s for 1024 × 1024 pixels. All-galvo scan imaging is superior to cumulative GR scan imaging to obtain GT data because multiframe averaging most likely leads to blurring, slight degradation of the resolution, and lack of fine texture details. The pixel number of the input images was a quarter of that of the GT images to accelerate the acquisition. To simultaneously collect structural information (noncentrosymmetry) from the SHG signals and functional information from the 3PA NADH and 2PA FAD signals, we tuned the excitation wavelength of the femtosecond laser (~100 fs) to 1140 nm^4^. The laser beam was directed through the scan mirrors, scan lens, tube lens, and subsequently to the back focal plane of a 0.75-NA microscope objective. A precompensation for group delay dispersion (GDD) of 8,000 fs^2^ was applied to ensure a low power of <50 mW excitation. The autofluorescence and SHG signals were then collected and spectrally separated by the combination of dichroic mirrors (DM) and bandpass (BP) filters (see the section “Materials and methods”).

To resolve the incompatibility between high speed, large area and high resolution, high contrast, we proposed the deep neural network architecture (see the “Materials and methods” section; Fig. S1) to fast transform large-field inferior images to denoised superior images (Fig. 1a). Previous to this, due to the noncollinearity of the two scanning systems, we implemented an efficient image preregistration method^29^ to achieve fore alignment between the input images and the GT images (Fig. S2a and Note S1). The preregistered paired images thereby formed the training dataset. Then, we proposed a self-alignment pyramid, cascading, and deformable convolutions (SAPCD) framework (Figs. S2b, S3, and Note S2) based on feature extraction and alignment^30^. This framework was embedded in super-resolution networks to automatically learn and realize pixelwise alignment between the preregistered input and GT images. Without the SAPCD for adaptive convolution, the fine textures cannot be well resolved due to the misalignment of pixel locations, resulting in out-of-focus images compared to the input bicubic results with resolved details (see the ablation study in the section “Materials and methods”). Referring to the perceptual-driven residual-in-residual dense block in the enhanced super-resolution generative adversarial networks (ESRGAN)^31^, we proposed the residual-in-residual dense attention block (RRDAB) as the basic generator block (Fig. S4a, b and Note S4). Benefiting from the dense connections and feature attention, RRDAB has a higher capacity for improving image quality and resolution while retaining real features than the original residual block in the ESRGAN. In particular, the integrated channel attention mechanism can explicitly model the feature map interdependencies, and the spatial attention mechanism can unscramble the interspatial relationship of the feature regions (Fig. S4b) within the residual blocks for feature recalibration. For the discriminator (see the “Materials and methods” section and Fig. S1), we combined spectral normalizations^27,28^ to stabilize the GAN training. We also introduced the perceptual loss function (Fig. S4c and Note S4) based on high-level features extracted from the pretrained VGG19 networks^32^ to increase convergence speed and better reconstruct fine details and edges.

### Deep learning-enhanced label-free large-field imaging

Nonlinear optical imaging can provide abundant cancer invasion-associated information (by SHG) and redox-ratio information (by NADH and FAD) for comprehensive pathological analyses of cancers^4^. To demonstrate DLAM on transformation from high-noise, low-resolution autofluorescence-harmonic images to high-quality, high-resolution images, we extracted unstained samples from human ovarian cancer tissues. Thirty-two frozen sections with 5-μm thickness were obtained using a freezing microtome. We performed multifield nonlinear imaging on these slices with the GR and DG scanning systems, corresponding to the low- and high-resolution domains, to construct the training dataset. Each stitched image from either domain was split into small image tiles to reduce the memory requirements and accelerate the training and testing processes (see the “Materials and methods” section).

**Fig. 2:**
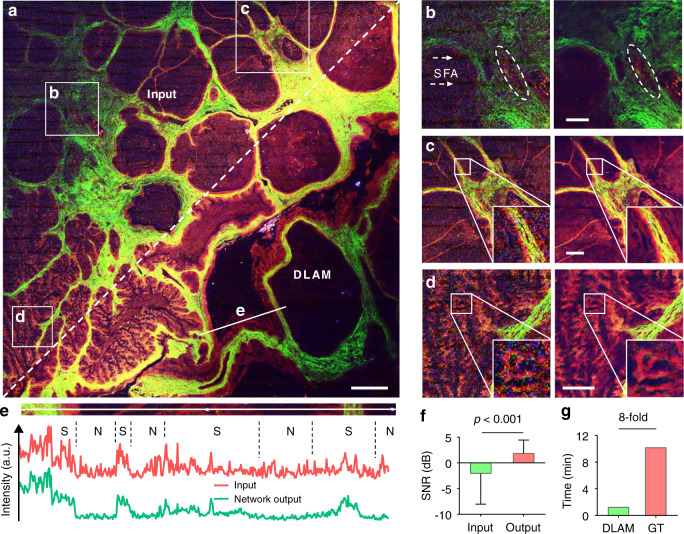
Deep learning-enhanced autofluorescence-harmonic imaging. **a** A 5.4 × 5.4 mm^2^ area image of human ovarian borderline carcinoma merged by 3PA of NADH (blue), SHG of collagen (green), and 2PA of FAD (red). These pseudocolor presentations are applied consistently throughout the paper. The white dashed line separates the registered input image and network output image. White squares indicate ROIs magnified in (**b**)–(**d**), which compare the registered input (left) and network output (right). White dashed arrows in **b** indicate the SFA. White ellipses indicate the blood vessel. **e** Intensity profiles are along the white solid line in (**a**). Black dashed lines separate the signal (S) and noise (N) regions according to the ROI above. **f** SNR of the raw input and network output images obtained using the GT as ideal images. A two-tailed Wilcoxon matched-pairs signed rank test was applied between the input and output images. *n* = 12 test large images. **g** Acquisition and inference time of DLAM compared to GT acquisition time. Scale bars, 500 μm in a and 200 μm in (**b**)–(**d**).

A comparison between the registered input image with 2176 × 2176 pixels and the network output image with 8704 × 8704 pixels is shown in Fig. 2a (see the whole input, output, and GT image with three nonlinear optical channels in Figs. S5–S7). Overall, the semantic information, especially the pathological features of ovarian borderline carcinoma, was well preserved at the network output. We identified the representative structures of mucinous ovarian cancer (MOC) at the top half of Fig. 2 and the micro-glandular or papillary architectures of high-grade serous ovarian cancer (HGSOC) at the bottom half of Fig. 2a. The atretic follicles and vessels in the MOC and HGSOC were surrounded by cancer-associated collagen (CAC) networks (indicated by SHG), which, however, were blurry in the input image. Regions of interest (ROIs) in Fig. 2a are shown in Fig. 2b–d, revealing that the “melted” fiber structures, vague filaments and erythrocytes, and noisy micropapillary reticulate architectures were clearly distinguished by deep learning inference. These denoised small-scale features by DLAM can be used for further pathological analyses and research purposes. For instance, it is difficult to determine the orientation of collagen fiber proliferation^33^ in the corrupted and low-resolution input images, calculated by the collagen fiber angles relative to the epithelium, due to the blurry textures. However, these orientations were explicit in the network output and GT images. The blurry textures also led to an incorrect aspect ratio^34^ (which indicates anisotropy of the extracellular matrix) of the fitting ellipse of the fast Fourier transform form of the input images, while the aspect ratio of the network output images was consistent with that of the GT images. These results demonstrate the difficulty in characterizing and diagnosing diseases using corrupted and low-resolution images, which was overcome using the reconstructed images enhanced by the networks. Another interesting finding is that the captured input images exhibited strong scanning fringe artifacts (SFA, i.e., the wide fringes in the left panel of Fig. 2b–d, also see Fig. S9) resulting from the fast GR scanning and stitching lattice artifacts (SLA, see Fig. S9) caused by the multifield stitching. These interferences, appearing more likely in the weak signal areas, were highly suppressed by the proposed deep network.

Additionally, the intensity profiles along the papillary tissue, CAC arrangement, and background area in Fig. 2a are given in Fig. 2e. The bottom profile for the input image shows a low distinguishability of noise and informative signals, which results in difficulty in semantic information extraction for the whole image. Nevertheless, these undesirable distortions were significantly reduced with good preservation of tissue structure features after reconstruction. For verification, we calculated the signal-to-noise ratio^23,35,36^ (SNR, see Fig. 2f) for the whole image. The network enabled a great improvement of SNR from −2.1 ± 5.9 to 1.9 ± 2.5 dB on average across 12 large-field images. Especially, the maximum SNR increase reaches 10.6 dB for a large 3PA image due to the high noise reduction and contrast improvement after deep learning. The SNR improvement demonstrates the ability of the network in minimizing the mix of noises, including the resonant SFA, Gaussian thermal noise, and shot (Poisson) noise, and background, such as SLA.

It should be mentioned that the acquisition time using the GR scanning system, *t*_input_, was 54 s, and the computational time using tribatch processing for the three nonlinear modalities, *t*_infer_, including the processing time spent reading a large image into memory, took less than half that time. In contrast, the time to capture a sufficiently high-quality image, *t*_GT_, reached 10 m 14 s (Table S1), which suggests that the inference allows a 24.3-fold reduction in acquisition time (*t*_infer_ compared to *t*_GT_−*t*_input_). Considering the GR acquisition time, DLAM allows an 8-fold imaging speed up (*t*_input_ + *t*_infer_ compared to *t*_GT_, Fig. 2g). This acceleration is significant since nonlinear optical microscopes are notoriously slow and can be further boosted using better GPUs and the direct transfer of large datasets from DAQ to GPU.

### Statistical analysis of image quality improvement

**Fig. 3:**
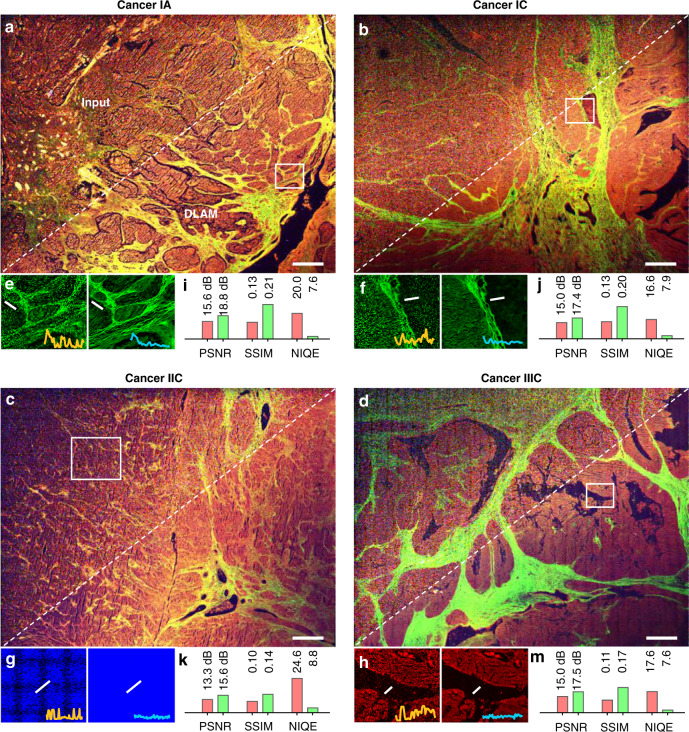
Label-free multimodal nonlinear images of human ovarian carcinomas. **a**–**d** Full view of the large field (5.4 × 5.4 mm^2^) at FIGO Stage IA, IC, IIC, and IIIC, with the white dashed lines separating the registered input (top left) and the network output (bottom right). White squares indicate ROIs magnified in (**e**)–(**h**) (left: input, right: output) for the highest metric enhancement. The white solid line in each inset image refers to the line of the shown cross-section. **i**–**m** Quality metrics for the input (pink) and output (pale green) large images are compared in the histograms. Scale bars, 500 μm.

We also applied the proposed deep network to human ovarian carcinomas with different International Federation of Gynecology and Obstetrics (FIGO) stages to demonstrate the additional benefits of using DLAM statistically. Figure 3a–d shows the fast label-free multimodal images of the clinically acquired tissue samples and the corresponding transformation results. The major features of HGSOC and MOC, including CAC fibers and networks, glandular or papillary architectures, and cancer-affected atretic follicles and vessels, were well distinguished in the reconstructed images (see the whole input, output, and GT images in Fig. S10). Fluctuations in the intensity profile of the input images were greatly reduced, while morphological information was retained and denoised, as demonstrated by the cross-sections in Fig. 3e–h. SLA in the 3PA channel in Fig. 3g was removed by deep learning inference (see more example illustrations in Fig. S9).

To quantify this quality improvement, we calculated the full-reference quality metrics comparing the input and output images at the pixel level concerning the pristine GT images and the no-reference quality metrics comparing their perception of quality. The perceptual features were trained on a database of the GT image modalities (see the section “Materials and methods”). The metrics shown in Fig. 3i–m, to some extent, indicate the reconstruction quality and prediction accuracy of the DLAM images. The average peak signal-to-noise ratio (PSNR), structural similarity index (SSIM), and natural image quality evaluator (NIQE) for the large-field (5.4 × 5.4 mm^2^) images with 8704 × 8704 pixels exhibited an increase of 2.6 dB, 52%, and 59% after the network restoration. These overall increases are moderate due to the variation in the signal (noise) strength over the millimeter-level scanning range, i.e., the large image contains both strong and weak signal areas. To better demonstrate the noise and distortion suppression capability of the network, we split the images into small tiles with 1088 × 1088 pixels and calculated the image metrics for those high noise level regions, as given in Fig. S11. The PSNR, SSIM, and NIQE on average across more than 173 image tiles exhibit an increase of ~4.5 dB, 79%, and 74%, respectively. This great enhancement verifies the strong capability of the RRDAB modules to suppress severe distortions, including noise, blurring, and artifacts. The perception-based image quality evaluator (PIQE), which is opinion-unaware and unsupervised, exhibits a low ability to quantify the improvement (38%) because it did not use pretrained features extracted from the GT images.

Further segmentation of the large images into smaller tiles (512 × 512 pixels) to obtain the noisiest areas demonstrates a maximum quality improvement of 13.3 dB for PSNR in the SHG channel, 316% for SSIM in the 3PA NADH channel, and 97% for NIQE in the 2PA FAD channel. Interestingly, these maximum quality enhancements were achieved in different optical modalities, which suggests the necessity of evaluating multimodal image reconstruction with diverse standards. We also evaluated the output results using the mean-square error (MSE) and blind/referenceless image spatial quality evaluator (BRISQUE), as detailed in Note S5. Their values again confirm the superior enhancements by the proposed deep network. Therefore, DLAM enables image reconstruction with prominent similarity to the GT in contrast, luminance, perception quality, structure, and variance.

### Transfer learning to skin tissues and resolution enhancement

**Fig. 4:**
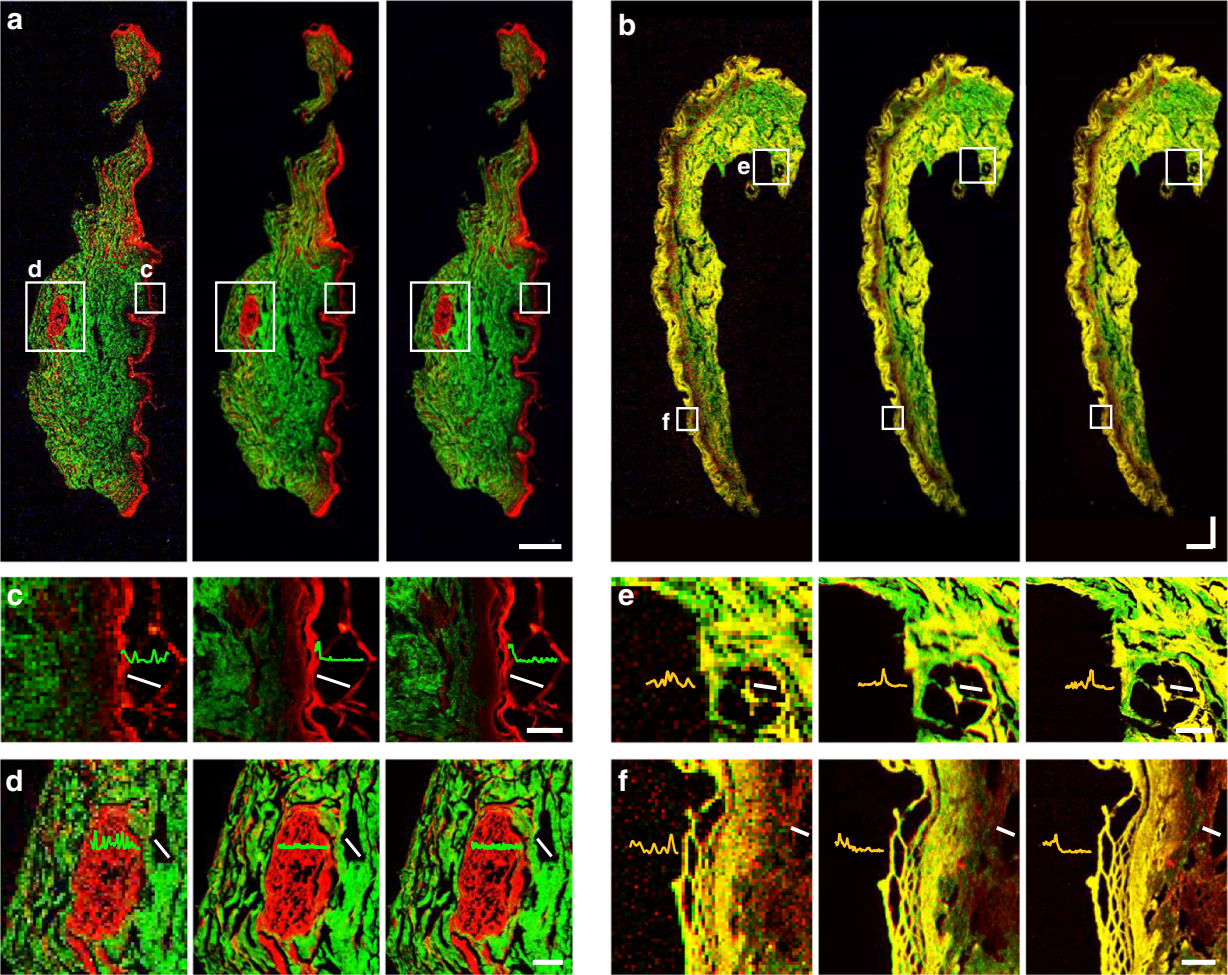
Label-free multimodal nonlinear images of human skin. Large-field images of normal skin tissue (**a**) and pigmented nevus tissue (**b**) with ROIs (white squares) magnified in (**c**)–(**f**). From left to right: registered input, network output, and GT images. White solid lines in **c**–**f** refer to the line of the shown cross-section. Scale bars, 500 μm in (**a**), (**b**), 50 μm in (**c**), (**f**), and 100 μm in (**d**), (**e**).

We further demonstrated the transfer learning capability of DLAM by transforming the captured raw images of human skin pathological tissues to match the corresponding GT images (Fig. 4). Despite the weak and indistinct 3PA NADH signals on the skin tissues, after transfer learning, the deep network dramatically reduced the distortions and noise and improved the quality of the input GR scanning images. The epidermis, mainly containing suprabasal keratinocytes indicated by 2PA FAD, and the dermis, mainly containing collagen fibrils, microfibrils, and elastic fibers indicated by SHG, were reconstructed more clearly, providing very good agreement with the GT images. Detailed features of keratin intermediate filaments in Fig. 4c, small tubular structures of sweat glands in Fig. 4d, and dense irregular connective tissue in the reticular region in Fig. 4e, f were clearly resolved at the network output. Noise fluctuations shown in the intensity profiles in the input images were highly suppressed by the deep learning inference, producing in-focus informative features approaching those of the GT images. These more elaborate details are attributed to the resolution enhancement by the embedded super-resolution framework. To quantify the resolution enhancement, we captured pristine GT images using an oil-immersion 1.4-NA objective and downsampled these images as the input of the network to learn high-resolution images. The results are summarized in Note S6 and Fig. S12, where the mean FWHM of the input point-spread function (PSF) is centered at ~481 nm, far above the FWHM of the SHG microscope PSF. The mean FWHM of the PSF of the network output approaches the PSF results of the GT, with a mean FWHM of ~289 nm versus ~282 nm, respectively (Fig. S12b, c). We also calculated the Fourier ring correlation (FRC) from the large SHG images (Fig. S12d), which is less prone to subjective bias and measurement errors^37^, as shown in Fig. S12e. The result further verified the resolution improvement after learning. Therefore, the proposed network provides a high improvement in spatial resolution, allowing the system to discern more precise textures and details. With such resolution enhancement, DLAM can reveal collagen fiber orientation and arrangement for clinicopathologic analyses^3,4^.

### Prevention of reconstruction artifacts

**Fig. 5:**
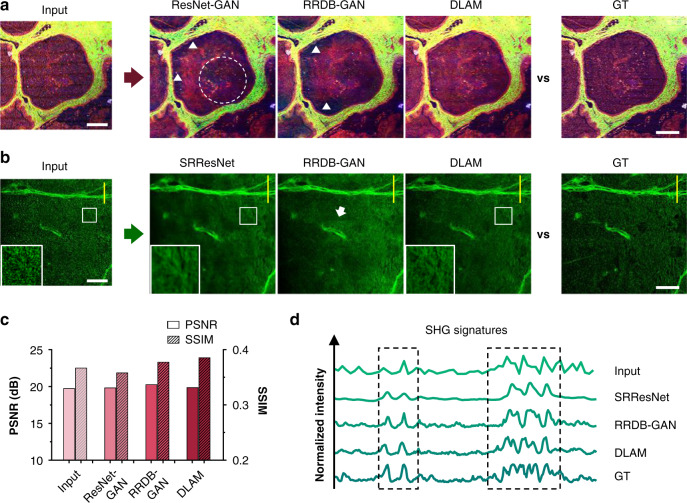
Comparison of DLAM results against other deep networks. Additionally, see Figs. S13 and 14. **a** Label-free multimodal images and corresponding reconstruction images of a carcinomatous atretic follicle. White dashed circles and triangles indicate slight and obvious reconstruction artifacts, respectively. **b** SHG (with 3PA FAD crosstalk^4^) images of collagen fibers and carcinoma cells. White squares indicate ROIs magnified in the insets. The white arrow indicates the discontinuity in the stitching. **c** PSNR and SSIM for different network outputs. **d** Intensity profiles along the yellow lines in (**b**). Black dashed squares indicate the SHG signatures. Scale bars, 300 μm in a and 150 μm in (**b**).

GAN reconstruction is essentially an ill-posed (inverse) problem^38^ and is prone to reconstruction artifacts when provided with inadequate training data^25^. Under submicrosecond pixel exposures, autofluorescence images of biological tissues are susceptible to obstinate noise derived from high-speed sampling and image-detection devices, including the resonant SFA, readout noise, dark current, and shot noise. When the standard deviation (STD) of noise (e.g., ~20 at 8-bit RGB) exhibited a similar amplitude to that of the average signal (e.g., ~62) in the input images, typical GAN frameworks, e.g., ResNet- and RRDB-GAN, began to show random, noncontinuous artifacts (Fig. 5a). Although these networks with adversarial loss can infer high perceptual quality images (see the no-reference quality metrics in Table S2), the deceptive artifacts reconstructed, i.e., the incorrect tissue features in Fig. 5a, were obvious. These highly plausible artifacts hallucinated by the networks can be challenging to detect in the absence of contradictory information (e.g., in unsupervised learning)^25^. For some GAN-free networks (such as SRResNet with simple MSE loss^27^) that can easily attain a high score in full-reference quality metrics (Table S2), the output images remain considerably out of focus (see the ROI magnified in the insets of Fig. 5b). This network fails to achieve a good tradeoff between noise and blurring due to overdenoising and a lack of high-frequency information. DLAM with channel and spatial attention, which can focus the important feature maps and their useful regions, avoids blurring and greatly improves the fidelity and quantitative nature of the super-resolution reconstructions compared to the conventional GAN frameworks (Fig. 5a, b).

We also quantified the reconstruction distortions of DLAM outputs by computing the resolution scaled error (RSE), resolution scaled Pearson coefficient (RSP), and error maps^15^ (see the “Materials and methods” section). The analysis in Fig. S14 reveals that DLAM did not generate noticeable reconstruction artifacts or blurring. The DLAM output results had a lower level of spatial mismatch error than the other network outputs concerning the input image (see the error maps in Fig. S14) and agree well with the GT image (Fig. 5a). To further confirm the conclusion, we overlapped the output image of different networks and the GT image in two complementary colors (Fig. S14). The merged images reveal that DLAM has no visualized transformation deviations compared to the other networks with respect to the GT image, which brings high credibility to the RRDAB reconstruction. The same conclusion applies to other test images. Additionally, since large images are usually split into contiguous tiles to reduce storage requirements (4 × 4 segments in this work, see the “Materials and methods” section), the independent inference for each split tile might cause discontinuities in the stitching (indicated by the white arrow in Fig. 5b). However, this artifact, as well as blurring, also were not observed in the resulting images using RRDAB.

Notably, the above mistakes made by other deep networks do not result in a significant reduction in full- and no-reference quality metrics (Fig. 5c and Table S2) in contrast to DLAM (e.g., the difference <2% for PSNR and <7% for SSIM). These networks have decent noise suppression in weak or no signal areas, yet their reconstruction distortion or oversmoothing is discernible in the SHG signatures in the intensity profiles given in Fig. 5d. In contrast, DLAM produces more distinct, realistic images, resolving the CAC filaments, which are covered by substantial speckles in the input images, compared to the pristine references (Figs. 2b–d and 5c). Therefore, DLAM can restore high-quality approximations of multimodal nonlinear images from high-speed sampling compared to typical GAN reconstructions and predict authentically where plausible details appear likely.

## Discussion

GR and DG scanning are the two major laser scanning methods for optical microscopes. We pursue the high speed of the former while requesting the high resolution of the latter, which are often contradictory and alternative. By removing the limitations of sophisticated mechanical devices and optical setups, DLAM possesses the advantages of the two scanners and, hence, enables label-free, large-area, speed- and resolution-enhanced multimodal imaging. A common concern for deep learning reconstruction is the requirement of thousands of training images. However, we reduced it to a mere 24 large pairs using multifield scanning and automatic stitching to avoid cumbersome procedures and long waits. With a conveniently obtained database, a two-photon excitation laser scanning microscope equipped with a GR scanner can turn into a galvo-quality imaging platform optionally to have more powerful functions and a wider application scope. Nevertheless, the network inference speed is still limited by the residual network depth. Recently, original and modified ResNet^39^ were proposed to solve high-level computer vision problems, including recognition, classification, and detection. Although it is obviously not optimal to directly apply the ResNet architecture to low-level computer vision problems such as super-resolution^39^, combining an appropriate mechanism (such as attention modules) to generate super-resolution images with high perceptual quality can be feasible. This may significantly increase the computational speed of the deep learning inference because ResNet exhibits more than six times faster than RRDB with heavy dense layers.

Different algorithms have been reported to improve the spatial resolution of optical microscopes, e.g., deconvolution (based on a PSF) reconstruction algorithms in structured-illumination microscopy (SIM)^36,40^ and quantum image scanning microscopy^41^. Additionally, some deep CNN or GAN frameworks have been reported to transform confocal or wide-field images to match the resolution acquired with a stimulated emission depletion (STED) microscope^15^, stochastic optical reconstruction microscope (STORM)^42^, and photoactivation localization microscope (PALM)^16^. These approaches demonstrated an approximately twofold increase in spatial resolution despite a micron FOV. Our method achieved a resolution improvement of the same magnitude at ×4 pixel magnification despite a GR scanning rate limit (usually 8–12 kHz) and a need for a hybrid scanner, which, in fact, is commonly equipped on recent commercial microscopes.

Increasing the excitation laser power to attain a higher SNR can help solve ill-posed inverse problems; however, this may lead to photobleaching and phototoxicity^40^. Many algorithms have been developed for artifact reduction and SNR improvement in medical imaging. For instance, a deep CNN to map low-dose CT images toward corresponding normal-dose CT images showed an ~0.5-dB improvement in PSNR^43^; another CNN combining multiresolution decomposition and residual learning to remove artifacts showed a 1–4 dB improvement in PSNR^44^; a recently reported task-aware compressed sensing with GAN for optimizing MRI imaging demonstrated a 2–3 dB improvement in PSNR^45^. Nevertheless, oversmoothing, out-of-focus, and deceptive artifacts arising in CNN or GAN framework reconstruction have not yet been comprehensively studied for deep learning-enhanced laser scanning microscopy. Through the integration of RRDAB and high-level perceptual loss, DLAM did not generate noticeable reconstruction artifacts and distortions and hence achieved authentic and realistic outputs, with a maximum PSNR increase of 13.3 dB (average 4.5 dB). The reconstructed high-quality images and the GT images are similar in terms of low-level pixel values, high-level abstract features, and the overall concept and style.

In summary, with the proposed efficient deep network architecture and conveniently obtained training dataset, DLAM highly suppresses SFA, SLA and other noises and overcomes the distortion problems for high-speed label-free imaging. Additionally, it offers a solution to the time consumption problem for high-quality and large-field image acquisition. Outputs of the microscope can be greatly improved in a small computational time without a design of redundant optical paths or upgrading of the device and hardware of the imaging platform. We demonstrated the applicability of the network by accelerating image acquisition and post-processing steps that can leverage higher image quality and visualize finer microstructure of clinicopathologic ovarian and skin tissues. Shortening the turnaround time with enhancement in spatial resolution enables rapid, large-field, stain-free histopathology of tissue specimens that can possibly supersede surgical frozen section analysis. The restored results can be used for better quantification of tumor-associated collagen signature in the extracellular matrix^33^, metabolic analysis involving FAD and NADH for cancer diagnosis^46^, and cell and extracellular component segmentation for revealing the complexity and heterogeneity of the tumor microenvironment^47^. Furthermore, in addition to the high-speed, high-quality, and high-fidelity reconstruction of autofluorescent-harmonic images of unstained pathological tissues, DLAM can also be applied to brain structure and function investigations without genetically encoded calcium indicators (GECIs), such as GCaMP. Future exploration will push the spatiotemporal limits of DLAM for high-speed super-resolution cell structure analysis, brain 3D in toto observation, and in vivo diagnostic examination, which can help facilitate applications of optical microscopes in biomedical research and clinical diagnosis.

## Materials and methods

### Optical setups and image acquisition

The multiphoton inverted microscope was equipped with a GR scanner for high-speed imaging and a DG scanner for high-resolution imaging. An autoalignment system can collimate the laser beam rapidly when the BS with motorized shutters switched the beam between the two scanners. An excitation femtosecond laser with a pulse width of ~100 fs and a repetition rate of 80 MHz (Chameleon Discovery, Coherent) was applied with a GDD precompensation of 8000 fs^2^ and directed to the apochromatic objective (MRD70200, ×20, 0.75 NA, Nikon). This precompensation ensured a low power of <50 mW at 1140 nm excitation to minimize photochemical and thermal stress and image distortion, while the photodamage at a typical long wavelength excitation (1080–1180 nm, 80 MHz, 100–250 fs, 3.3 μs px^−1^) was reported to be 120 mW^46,48,49^. Moreover, the fast-scanning mode with a small pixel dwell time can mitigate the total energy deposited in the samples. This can be an advantage of our method because it transformed the inferior images captured by the fast resonant scanning mode to the high-quality images approaching the GT images with a large pixel dwell time. Thus, our deep networks help to alleviate photochemical and thermal stress on the samples. The backscattered laser and emission signals were separated by a VIS/IR DM. The emission autofluorescence and harmonic generation signals were spectrally separated by the filter combination: (1) LP 488 nm and BP 450/50 nm for 3PA NADH, (2) LP 593 nm and BP 570/10 nm for SHG (with concomitant 3PA FAD), and (3) LP 685 nm and BP 641/75 nm for 2PA FAD. The FOV of a single image was 634.88 µm × 634.88 µm.

The trichromatic channels of the images were constructed by three nonlinear modalities. A single image consists of 256 × 256 pixels for the input and 1024 × 1024 pixels for the GT, while a large image consists of 2176 × 2176 pixels for the input and 8704 × 8704 pixels for the GT. These captured large images were formed with 16 × 16 scan fields and stitched by blending with 50% overlap to minimize the stitching traces. The acquisition time for the input and GT images, as well as the reconstruction time for the deep learning, are shown in Table S1. A large input image with the three channels was obtained in 54 s using the GR scanning mode operating at 30 fps with a pixel dwell time of 0.5 μs. A large GT image with the three channels was obtained in 10 m 14 s using the DG scanning mode at 0.474 fps with a pixel dwell time of 2 μs (i.e., the parameters usually applied to obtain a high-quality image). These acquisition times for the large images included the movement of the mechanical stage and stitching of multiple fields. The pixel resolutions for the input images and the GT images were 2.49 and 0.62 μm px^−1^ (document calibration), respectively.

### Sample preparation

Ovarian and skin tissues were collected from patients at China–Japan Union Hospital of Jilin University and The Sixth People’s Hospital of Shenzhen, respectively, with approval of biomedical research ethics involving humans by the Scientific Research Ethics Committees. All patients with diagnosed ovarian or skin tumors were approached for recruitment. Physicians recruited patients and obtained study consent. Prospective enrollment began on June 1, 2019, and closed on March 1, 2021. Experienced gynecological oncologists conducted histological identification and classification according to the FIGO classification standards. Tissue samples were surgically removed and snap-frozen in liquid nitrogen and stored at –80 °C until being cut into 5-μm sections for unstained applications using a freezing microtome (CM1850, Leica, Germany). The frozen tissue sections were simply covered with a coverslip, imaged by multiphoton microscopy, and preserved by formalin (Anatech) fixation and paraffin embedding.

### Deep neural network architecture

The basic framework of the generator network and discriminator network used in this work are shown in Fig. S1. Compared to DG imaging, GR imaging usually has a mass of noise and fringes; thus, the luminance adjustment algorithm^50^ is not suitable for this work. Due to the noncollinearity of the two scanning systems, an efficient image registration method should be developed because the training pairs (raw input and GT images) were rather mismatched. We implemented a well-known feature extraction and warping method, termed ORB^29^, short for oriented FAST (features from accelerated and segments test) and rotated BRIEF (binary robust independent elementary feature). The ORB algorithm (see Fig. S2a and Note S1) can greatly reduce the spatial mismatch of the coupled pixels to form the training dataset. However, the resulting warped (preregistered) images are still not completely aligned with the GT images. Therefore, we proposed the SAPCD module (Figs. S2b, 3 and Note S2) to calibrate feature maps of different scales and attained further alignment between the warped input images and the GT images. This module embedded in the networks can automatically learn to optimize the pixelwise alignment. Then, we kept the residual-in-residual connections of ESRGAN^31^ and developed the RRDAB modules (Fig. S4a and Note S3) for super-resolution reconstruction. The RRDAB modules employ a more complex structure than the original RRDB block in ESRGAN to reconstruct the registered images with the previously calibrated features. A channel attention module, squeeze-and-excitation networks (SENet)^51^ that can direct the networks to select proper feature maps, and a spatial attention module (SAM)^52^ that can indicate the feature barycenter were introduced to further guide the reconstruction processes. They help to identify the crucial features and feature regions to improve super-resolution details and avoid oversmoothness. The attention-guided dense connections (Fig. S4b) prevent the super-resolution reconstruction from generating deceptive artifacts with the assistance of the proper loss functions. Note that embedding the attention modules in the existing network can lead to a small increase in additional parameters and calculations (Table S1). The resolution for the input images is improved using two ×2 nearest interpolation with convolution for ×4 upsampling. After RRDAB reconstruction, the quality of the images can be significantly boosted to high quality compared to the original inputs. Finally, referring to EdgeConnect^53^, we added spectral normalizations to the discriminator to stabilize the GAN training by, in effect, bounding the Lipschitz constant of the discriminator function (Fig. S1b). We used high-level perceptual loss in the pretrained VGG19^32^ as the feature extraction network to clarify the edges and textures of the generated images (Fig. S4c and Note S4). The introduced perceptual loss ensures pixelwise identity while avoiding oversmoothing occurring in other super-resolution methods (e.g., SRResNet and SRGAN-MSE^27^).

### Training and testing details

We removed part of the border (16 pixels on each side) of the 2176 × 2176-pixel input images that had been roughly registered after ORB and then cropped the images into small tiles at a pixel size of 128 × 128 and a step size of 64. These pixel ranges and steps ensure enough pixel overlap between the adjacent tiles. The GT image was cropped into small tiles at 512 × 512 pixels. Considering the memory capacity, we set the batch size to eight during training. In each epoch, we randomly selected a GT image tile at 256 × 256 pixels and an input image tile at 64 × 64 pixels. We randomly selected one of up, down, left, right flip, and [0, 90°, 180°, 270°] rotations for data augmentation.

The weight of high perceptual loss in loss is *λ*_1_ = 0.1, and the weight of GAN loss is *λ*_2_ = 0.05. In the reconstruction module, we chose adaptive moment estimation (Adam)^54^ as the optimizer of the generator and discriminator, *β*_1_ = 0.9, *β*_1_ = 0.99. The generator and discriminator were alternately updated until the result converged to a plateau.

In the model training, we used preregistered input images and nondownsampling GT images from 24 large-scale paired images, corresponding to 39,204 segmented pairs for each nonlinear modality. We trained the network with 400,000 iterations on the PyTorch framework using a GTX 1080TI GPU (11 GB memory). In the prediction, the large input images were divided into 4 × 4 = 16 tiles due to video memory limitations. To utilize full resources, we used three GTX1080TI graphics cards, where each GPU had a batch size of one, corresponding to one of the nonlinear modalities (RGB channels), to accelerate the inference processes. Training and testing had no data overlap, i.e., the test images shown in this article were blindly generated by the deep network.

### Ablation study

To understand the contribution of the components to the performance of DLAM, we performed an ablation study. First, we used the pretrained ESRGAN architecture^31^ with the GT images and synthetically downsampled GT ↓ 4 images as the paired training dataset. However, this network failed to remove real obstinate noise with full statistical complexity (Fig. S15). These noises, substantially existing in the low-resolution images, precluded the generation of high-resolution textures because the synthetic images degraded from the GT images were essentially different from the captured data. Therefore, we next trained the network with the real source and GT data acquired by the microscope.

Without preregistration by ORB, the position deviation of pixels between the input images and the GT images varied from 0 to 200. Since these images were cropped into small tiles for training, as a consequence, there may exist insufficient overlaps of the cropped ROIs of the input images and the GT images. The training process was thereby difficult to converge, and the resulting images were exceedingly nebulous, as shown in Fig. S15.

Without the SAPCD for adaptive convolution, the RRDB still has a sufficiently large receptive field to capture the pixel deviations. Thus, the overall outline of the resulting image is clearer than the above (see the magnified ROI in Fig. S15). However, the fine textures and details were barely reconstructed due to the limitation of the network fitting ability. The pixel positions were inaccurately calibrated, resulting in out-of-focus images compared to the input bicubic results with clear details.

The GAN result was obtained by combining ORB, SAPCD, and RRDB with L1 loss instead of perceptual loss, as the discriminator was boosted in recovering buried high-frequency details. However, minimizing L1 encourages the search for pixelwise averages of plausible solutions (although better than MSE), resulting in a strong blur and poor perceptual quality with fuzzy and false textures (see the corresponding magnified ROI in Fig. S15).

Adding high-level perceptual loss gives clearer fine textures. The resulting image generated from RRDB-GAN was more natural and close to the GT image in pixel values and abstract features (Fig. S15). The ability of the preregistration and registration methods can be demonstrated by comparing the third-row images with more distinct textures to the second-row images in Fig. S15. Finally, the introduction of the attention modules into DLAM prevented the deep networks from making obvious mistakes and, hence, promoted the generation of authentic results.

### Benchmarks

We compared our network architecture with optimized SRResNet, ResNet-GAN^27^, and RRDB-GAN^31^ to demonstrate its competitive performance. The training data for these networks were all registered by ORB feature extraction and the SAPCD module to realize pixelwise self-calibration. SRResNet was built based on the reported model with MSE loss optimized for our data. ResNet-GAN employed a 34-layer ResNet as the backbone network of its GAN generator. Following the enhanced deep residual networks for single image super-resolution^39^, we removed the batch normalization structure to obtain higher performance. The RRDB-GAN employed the state-of-the-art RRDB as the GAN generator backbone network. The network was optimized for perceptual loss, which is more stable to changes in pixel space^27^. The other parts of the RRDB-GAN networks can be found in the ESRGAN^31^. We used the same VGG layer, discriminator, and training dataset as our networks for these networks to reconstruct the input images with high perceptual quality.

### Data processing

The RGB color channels of the images were constructed by three nonlinear modalities. The input and GT images were produced in 8-bit TIFF files using commercial software (NIS-elements AR, Nikon) to reduce storage requirements and speed up data read, write, and transfer^22^. Autofluorescence and harmonic images with a relatively low contrast were regulated by adjusting the dynamic ranges (brightness/contrast) in ImageJ to better display the indiscernible morphological features^4^. Additionally, the presented images were downsampled (without average, bilinear, and bicubic interpolation) to better show the consistency and difference between input, output, and GT. These adjusted dynamic ranges and downsampling were applied consistently for the input, output, and GT images when they were compared in the same panels throughout the paper.

### Image quality and resolution evaluations

The major full-reference quality metrics, MSE, PSNR, and SSIM, the major no-reference quality metrics, BRISQUE, NIQE, and PIQE, and the reconstruction artifact metrics, RSE and RSP, were calculated for each channel (nonlinear modality) of the images to quantify the transformation deviations. In particular, we trained a custom NIQE feature model extracting features from 46 large-field GT images containing different modalities and calculated the NIQE scores for the registered input images and network output images using the trained model. The globally averaged scores, RSE and RSP^15^, as well as the corresponding error maps, were calculated using NanoJ-Squirrel Plugin in Fiji to visualize the discrepancy between input, output, and GT images.

For evaluating spatial resolutions, we used a high NA objective (MRD71600, ×60, 1.40 NA, Nikon) to capture 150 high-resolution images at a 570-nm emission and Nyquist sampling, and downsampled (scale: 0.25) these image as the input of the network for deep learning. We fit the cross-sectional profile of the extracted PSFs with a Gaussian function. These fittings attained 95% fitting confidence bounds for the input and output images: degree-of-freedom adjusted coefficient of determination (adjusted R-square) > 0.98, sum of squares due to error (SSE) < 0.02, and root mean squared error (RMSE) < 0.06. To reduce the measurement errors and subjective bias, we calculated the spatial resolution from the FRC histogram, which was formed by cross-correlating each bin divided from the spatial frequency spectra of two images^37^. The FRC resolution was defined as a cutoff frequency at which the cross-correlation value reaches a preset threshold (Fig. S12e).

The STD of noise was calculated over a large background, and the STD of the signal was calculated by averaging over a strong-signal area.

## Supplementary information

Supplementary Information

## Data Availability

We declare that the major data supporting the findings of this work are available within the manuscript and Supplementary Information files. All data used in this study are available from the corresponding author upon reasonable request. All custom codes used in this study are available from the corresponding author upon reasonable request. The code for network training and prediction is publicly available at https://github.com/Armstrong-lsw/SR_Self_Align.
