# Supplementary material for: Deep learning autofluorescence-harmonic microscopy
Source: Light Sci Appl. 2022 Mar 29;11:76. doi: 10.1038/s41377-022-00768-x (PMC8964717; doi:10.1038/s41377-022-00768-x)
Supplement: Supplementary file 1 — Supplementary Information [file 41377_2022_768_MOESM1_ESM.pdf]

Supplementary Information

Deep learning autofluorescence-harmonic microscopy

Binglin Shen[1†], Shaowen Liu[2†], Yanping Li[1], Ying Pan[3], Yuan Lu[4], Rui Hu[1], Junle Qu[1], Liwei Liu[1*]

[1]*Key Laboratory of Optoelectronic Devices and Systems of Guangdong Province and Ministry of Education, College of Physics and Optoelectronic Engineering, Shenzhen University, Shenzhen 518060, China.*

[2]*Shenzhen Meitu Innovation Technology LTD, Shenzhen, 518060, China.*

[3]*China–Japan Union Hospital of Jilin University, Changchun, 130033, China.*

[4]*The Sixth People's Hospital of Shenzhen, Shenzhen 518052, China*

[†]These authors contributed equally.

[*]Correspondence should be addressed to L.L. (liulw@szu.edu.cn).

Note S1 Preregistration for preparation of paired training dataset

Generally, there is a large misalignment between GR imaging and DG imaging due to the difference in scanning paths. It is difficult to manually align because this misalignment is an affine transformation including rotation and translation. And it is also difficult to align with the difference of pixel gray value because the pixels of the two images are quite different and there is a lot of noise in the resonant image. After testing, ORB[1] is more suitable for our data set than other feature extraction and matching operators such as scale invariant feature transform (SIFT)[2] and speeded up robust features (SURF)[3]. ORB has the advantages of insensitivity to illumination, scale consistency, and rotation invariance, thus, it is often used for feature matching to find optical flow between motion frames, image stitching, and other tasks.

(1) We first downsampled GT to the same size as input, denoted as HR↓4, and input as low-resolution (LR). Orb feature points include key points and descriptors, KP_{LR}, DES_{LR}, $KP_{HR\downarrow 4}$, and $DES_{HR\downarrow 4}$. For keypoint matching, Hamming distance was used as the descriptor distance metric for its corresponding descriptor, and the brute force matching method was used to obtain a preregistration feature point pair, as shown in Fig. S2a.

(2) There were some outliers in the feature point pair, we used the iterative optimization method to calculate the homography matrix of the affine transformation. Since there was only a rotation transformation R, and a translation transformation t, between the input and the HR↓4, thus,

$$\begin{bmatrix} x' \\ y' \\ 1 \end{bmatrix} = \begin{bmatrix} R & t \\ 0^T & 1 \end{bmatrix} \begin{bmatrix} x \\ y \\ 1 \end{bmatrix} \tag{1}$$

where (x_i, y_i) and (x_i', y_i') are the key coordinates in KP_{LR} and $KP_{HR\downarrow 4}$ after the preregistration, respectively.

$$R = \begin{bmatrix} \cos\theta & -\sin\theta \\ \sin\theta & \cos\theta \end{bmatrix} \tag{2}$$

θ is the rotation angle.

$$t = \begin{bmatrix} t_x & t_y \end{bmatrix}^T \tag{3}$$

is translation between (x_i, y_i) and (x_i', y_i'). Or, Eq. (1) can be expressed by

$$s[x', y', 1] = H[x, y, 1] \tag{4}$$

that is

$$s\begin{bmatrix} x_i' \\ y_i' \\ 1 \end{bmatrix} = \begin{bmatrix} h_{11} & h_{12} & h_{13} \\ h_{21} & h_{22} & h_{23} \\ h_{31} & h_{32} & h_{33} \end{bmatrix} \begin{bmatrix} x_i \\ y_i \\ 1 \end{bmatrix} \tag{5}$$

where H can be calculated by minimizing

$$\sum_{i=1}^{n}\left(\left(x_i^{'}-\frac{h_{11}x_i+h_{12}y_i+h_{13}}{h_{31}x_i+h_{32}y_i+h_{33}}\right)^2+\left(y_i^{'}-\frac{h_{21}x_i+h_{22}y_i+h_{23}}{h_{31}x_i+h_{32}y_i+h_{33}}\right)^2\right) \quad (6)$$

using convex optimization method[2], where n is the number of the key point pair, and $s=1$ represents the image scaling.

We then remove the interference of outliers using the random sample consensus (RANSAC) optimization algorithm[4] and used the Levenberg-Marquardt optimization method[5] to further improve the robustness during iterative optimization.

(3) We cropped the LR image into small image blocks because of graphics memory limitation and customarily, the receptive field of the low-level vision task network does not need to be large. A large area of regions of interest (ROI) overlap could still be obtained because the pixel misalignment between the LR and HR↓4 was very small due to the preregistration.

Note S2 Self-alignment pyramid, cascading, and deformable convolutions

Deformable convolution was proposed to learn irregular convolution positions to obtain more efficient feature expression capabilities[6] and shows good performance in object detection[7], recognition[8], semantic segmentation[6], video super-resolution[9], etc. The advantage of deformable convolution is that pixel calibration can be achieved at the feature level by selecting sampling point convolution with position deviation for multi-frame sequential images, without explicit motion estimation such as optical flow. Inspired by the video restoration framework with enhanced deformable convolution (EDVR)[10] which obtained better results than optical flow warping in video super-resolution, we used the modulated deformable module[11] as the single image pixel calibration module. In brief, given a convolutional kernel of K sampling locations, there are a weight and a pre-specified offset for the k-th location denoted as w_k and p_k respectively. For example, for a 3×3 kernel, $\mathrm{K} = 9$ and $p_k \in \{(-1,-1),(-1,0),\ldots,(0,1),(1,1)\}$. The aligned features at each position p (deformable convolution) are

$$x^a(p) = \sum_{k=1}^{K} w_k \cdot x(p + p_k + \Delta p_k) \cdot \Delta m_k \tag{7}$$

where Δp_k and Δm_k denote the learnable location offset and modulation scalar range [0,1] at the k-th location, respectively. $x(p)$ is the input feature maps at location p. Bilinear interpolation is applied in computing $x(p + p_k + \Delta p_k)$ as $p + p_k + \Delta p_k$ is mostly fraction. Thus, for both Δp_k and Δm_k, we used a separate convolution layer acting on the same input feature maps x with a total of 3K channels, where 2K channels for Δp_k, K channels for Δm_k with a sigmoid layer for range [0,1].

Referring to EDVR and inspired by the Laplacian pyramid super-resolution network (LapSR)[12] and texture transformer network for image super-resolution (TTSR)[13], we proposed SAPCD (Fig. S2b) based on pyramidal processing and cascading refinement. First, we performed feature extraction of Level 1 (L1, Fig. S3a). Then the feature of Li level (i = 2, 3, …) can be downsampled from that of L(i-1) level by stride convolution with stride equaling 2. At level Li, offsets (red boxes in Fig. S3b,c) are predicted with Li level features and ×2 upsampled offsets from L(i+1) level (purple dash lines in Fig. S3b,c). Similarly, aligned features (green boxes in Fig. S3b,c) are predicted with the deformable results and upsampled aligned features from L(i+1) level. That is

$$\Delta P^i = f\left(x, (\Delta P^{i+1})^{\uparrow 2}\right) \tag{8}$$

$$(x^{\mathrm{a}})^i = g\left(DConv(x^i, (\Delta P^i), ((x^a)^{i+1})^{\uparrow 2}\right) \tag{9}$$

where

$$\Delta P^l = \left\{\Delta p^l\right\} \tag{10}$$

f and g are different convolutions for different predicting tasks. $(\cdot)^{\uparrow s}$ stands for upscaling (bilinear interpolation) by a factor s. DConv is a deformable convolution given by Eq. (7).

Note S3 Residual-in-residual dense attention block for generator

Based on the residual-in-residual dense block (RRDB) module used in super-resolution generative adversarial networks (ESRGAN)[14], we proposed residual-in-residual dense attention block (RRDAB) as dense blocks to reconstruct the features after the SAPCD alignment (Fig. S4a). To enhance the dense connection that concatenates different receptive fields of input into channels, we used squeeze-and-excitation (SE) blocks, referring to SENet[15] and convolutional block attention module (CBAM)[16], adjacent to dense connection to learn channel and spatial attention. Networks with SE blocks can adaptively weight different fields, i.e., knowing which feature map to learn, and with spatial attention module (SAM) blocks following SE blocks can adaptively weight different regions, i.e. knowing which regions of the feature map to learn. Mathematically, we denoted F_i and F_{i-1} as input and output of the RRDAB module, denoted X and Y as input and output of residual dense attention block (RDAB), and denoted l_i as i-th layer in m-layer dense attention block (DAB) block and l_0 and l_m as the input and output layers, respectively.

$$DAB: l_i = \begin{cases} LReLU\left(Conv([l_0, l_1, ..., l_{i-1}])\right), i = 1, 2, ..., m-2 \\ Att\left(LReLU\left(Conv([l_0, l_1, ..., l_{i-1}])\right)\right), i = m-1 \\ Att\left(Conv\left([l_0, l_1, ..., l_{i-1}]\right)\right), i = m \end{cases} \tag{11}$$

where $[l_0, l_1, \dots, l_{i-1}]$ stands for the concatenation of the feature maps predicted in layers 0, 1, …, i–1. $LReLU$ is short for Leaky ReLU,

$$Leaky\ ReLU: y = \begin{cases} x, & if\ x \geq 0 \\ \alpha x, & if\ x < 0 \end{cases} \tag{12}$$

where constant $\alpha = 0.01$ in this paper.

As for the attention operation,

$$\begin{cases} Att: fea'' = SAM\left(SE\left(fea\right)\right) \\ SE: fea' = \sigma\left(FC\left(AvgPool\left(fea\right)\right)\right) = \sigma\left(FC_1\left(ReLU\left(FC_0\left(fea_{avg}\right)\right)\right)\right) \\ SAM: fea'' = \sigma\left(conv\left(fea'\right)\right) \end{cases} \tag{13}$$

where fea, fea_{avg}, fea', and fea'' denote input feature map, feature map after global average pooling, output feature map after SE block, and output feature map after SAM block, respectively. σ is sigmoid function.

$$AvgPool: fea_{avg} = \frac{1}{h \times w} \sum_{i=1}^{h} \sum_{j=1}^{w} fea(i, j) \tag{14}$$

where h and w are the height and width of fea, $fea \in R^{C \times h \times w}$, where C is the channel of fea. FC is the fully connected layer. $FC_0 \in R^{C/r \times C}$ and $FC_1 \in R^{C \times C/r}$, where r is the reduction ratio.

$$\begin{cases} RDAB: Y = X + DAB(X) \times \beta \\ RRDAB: F_i = F_{i-1} + RDAB\left(RDAB\left(RDAB(F_{i-1})\right)\right) \times \beta \end{cases} \tag{15}$$

where β is used for residual scaling in Fig. S4.

$$x^{\text{out}} = RRDAB_d\left(RRDAB_{d-1}\left(...RRDAB_2\left(RRDAB_1(x^{\text{a}})\right)\right)\right) \tag{16}$$

where x^{a} is the aligned feature from SAPCD. x^{out} is the output feature. d is the RRDAB cascading number.

$$I^{\text{HR}} = conv\left(L\mathrm{Re}LU\left(conv\left(\left(x^{fea1} + Att\left(conv(x^{\text{out}})\right)\right)^{\uparrow S}\right)\right)\right) \tag{17}$$

where S is the upsampling scale, which equals four in this work. x^{fea1} is the feature map after 1st conv layer in the feature extraction block in SAPCD, of whiich channel equals to that of x^{out}.

Note S4 Perceptual loss function for discriminator

Perceptual loss compares the high-level image feature representations (instead of pixel differences) extracted from GT and output to ensure their similarity of high-level information (content and global structure). We selected pretrained VGG19[17] as the feature extract network in the perceptual loss. After trying on feature layer $\varphi_{1,1}, \varphi_{2,2}, \varphi_{3,4}, \varphi_{4,4}, \varphi_{5,4}$ ($\varphi_{i,j}$ is the j-th feature map layer at the block before i-th max-pooling) which stand for low-to-high level vision feature, we chose $\varphi_{5,4}$ as perceptual loss layer for more distinct texture. Referring to perceptual loss in ESRGAN, we got the feature map before ReLU activation. Then, we applied a high perceptual loss as

$$L_{\text{hi-percep}} = \frac{1}{W_{5,4}H_{5,4}} \sum_{h=1}^{H_{5,4}} \sum_{w=1}^{W_{5,4}} \left(\varphi_{5,4}(I_{h,w}^{\text{GT}}) - \varphi_{5,4}(I_{h,w}^{\text{SR}}) \right)^2 \quad (18)$$

where $W_{5,4}$ and $H_{5,4}$ are the width and height of feature map $\varphi_{5,4}$ in VGG. I^{SR} is the generated super-resolution result, I^{GT} is the HRGT intensity.

Because of the application of GAN, the total loss for the generator is

$$I_{\text{Gen}}^{\text{SR}} = L_{\text{content}} + \lambda_1 L_{\text{hi-percep}} + \lambda_2 L_{\text{GAN\_gen}} \quad (19)$$

And the loss for the discriminator is

$$I_{\text{GAN\_dis}}^{\text{SR}} = -\log Dis_{\Theta\text{Dis}}(I^{\text{GT}}) + \log\left(Dis_{\Theta\text{Dis}}\left(Gen_{\Theta\text{Gen}}(I^{\text{LR}}) \right) \right) \quad (20)$$

where Gen and Dis are short for generator and discriminator, Θ_{Gen} and Θ_{Dis} are their network parameters. $\lambda_1$and λ_2 are coefficients of different losses.

$$L_{\text{GAN\_gen}} = \left(-\log Dis_{\Theta_{\text{Dis}}}\left(Gen_{\Theta_{\text{Gen}}}\left(I^{\text{LR}} \right) \right) \right) \quad (21)$$

is the generative loss that encourages the generator to make the super-resolution results natural that can deceive the discriminator. $L_{\text{GAN\_dis}}^{\text{SR}}$ discriminates the generated super-resolution result to false and GT to true. But they will converge after adversarial training, making the generator predict super-resolution images residing on the manifold of natural image[18], and visually, closer to the GT.

L_{content} is the L1 loss:

$$L_{\text{content}} = \frac{1}{WH} \sum_{h=1}^{H} \sum_{w=1}^{W} \left| I_{h,w}^{\text{GT}} - I_{h,w}^{\text{SR}} \right| \quad (22)$$

which ensures pixel-wise identical while avoids overly smooth in some super-resolution methods using mean-square error (MSE)[19].

Note S5 Comparison of image quality enhancement by different networks

Commonly used image quality indicators include MSE, PSNR, and the SSIM index. In most reconstruction tasks, these indicators can effectively evaluate image improvement. However, in superresolution reconstruction tasks, after the introduction of generative adversarial networks in recent years, researchers found that high PSNR or SSIM may not necessarily represent better reconstruction quality. This is because high MSE-based PSNR corresponds to excessive smoothing, while SSIM comprehensively evaluates luminance, contrast, and structure except for the perceived quality of the images. Although SRResNet attained the highest PSNR and SSIM, the reconstructed images tended to be overly smooth (Fig. 5b). ResNet- and RRDB-GAN networks had similar noise suppression capabilities to the DLAM but provided little small-scale information (Fig. 5d). The texture details in high PSNR or SSIM images do not necessarily meet human senses. Thus, we also used no-reference quality metrics, including BRISQUE, NIQE, and PIQE, to evaluate the image quality. Opinion-aware BRISQUE is limited to evaluating the same type of distortion and requires complicated custom differential mean opinion score values obtained through experimentation for the training datastore. It is difficult to reveal ambiguity (unless specially trained for such distorted quality-aware features). NIQE is opinion-unaware, related to local image sharpness[20], and refers to expected statistical features of the GT images. PIQE is opinion-unaware and unsupervised and can estimate block-wise distortion. These scores gave a large value (low quality) for the reconstruction ambiguity by SRResNet. Nevertheless, these metrics merely evaluate noise suppression and natural/perceptual quality improvement of ResNet- and RRDB-GAN networks despite a lost sight of reconstruction artifacts. The third set of indicators in Table S2, including the resolution scale Pearson correlation (RSP) scores and resolution scaled error (RSE) in Table S2, as well as the error maps in Fig. S14, reveal the difference in reconstruction artifacts reconstructed by different networks against the GT. Overall, DLAM guided by dual SE or SE-SAM modules attained a much lower RSE and higher RSP score due to its artifact and blurring prevention.

Note S6 Evaluation of resolution improvement

The embedded super-resolution framework in the generator (Fig. S1) has the capability to enhance the spatial resolution of a microscope. We extracted the PSFs of the label-free SHG microscope images and its downsampled results, as well as the network output images (Fig. S12a) to estimate their Rayleigh resolution[21]. The Gaussian FWHM[22-24] for the input image was calculated to be 501 nm, while the proposed deep network greatly improved it to 290 nm, which approached the value (275 nm) of the GT image (Fig. S12b). Nevertheless, axial resolution was not calibrated because pathological analysis of frozen sections is usually performed laterally and the tissue thickness is only 5 μm. To confirm overall resolution improvement by DLAM, we performed a statistical evaluation of the spatial resolution for the input, output, and GT images as shown in Fig. S12c. The mean FWHM for the input images was 481 nm. After the deep network learning, this FWHM was improved to 289 nm, providing a very good match to the PSF results of the SHG modality with a mean FWHM of 282 nm. Additionally, Fourier ring coefficient (FRC) has been reported to be an efficient “blind” resolution metric[25] which are less prone to prejudice in the selection process. We calculated the FRC on the SHG images (Fig. S12d) to yield an objective spatial resolution. The result demonstrates a resolution improvement of approximately 155 nm (Fig. S12e).

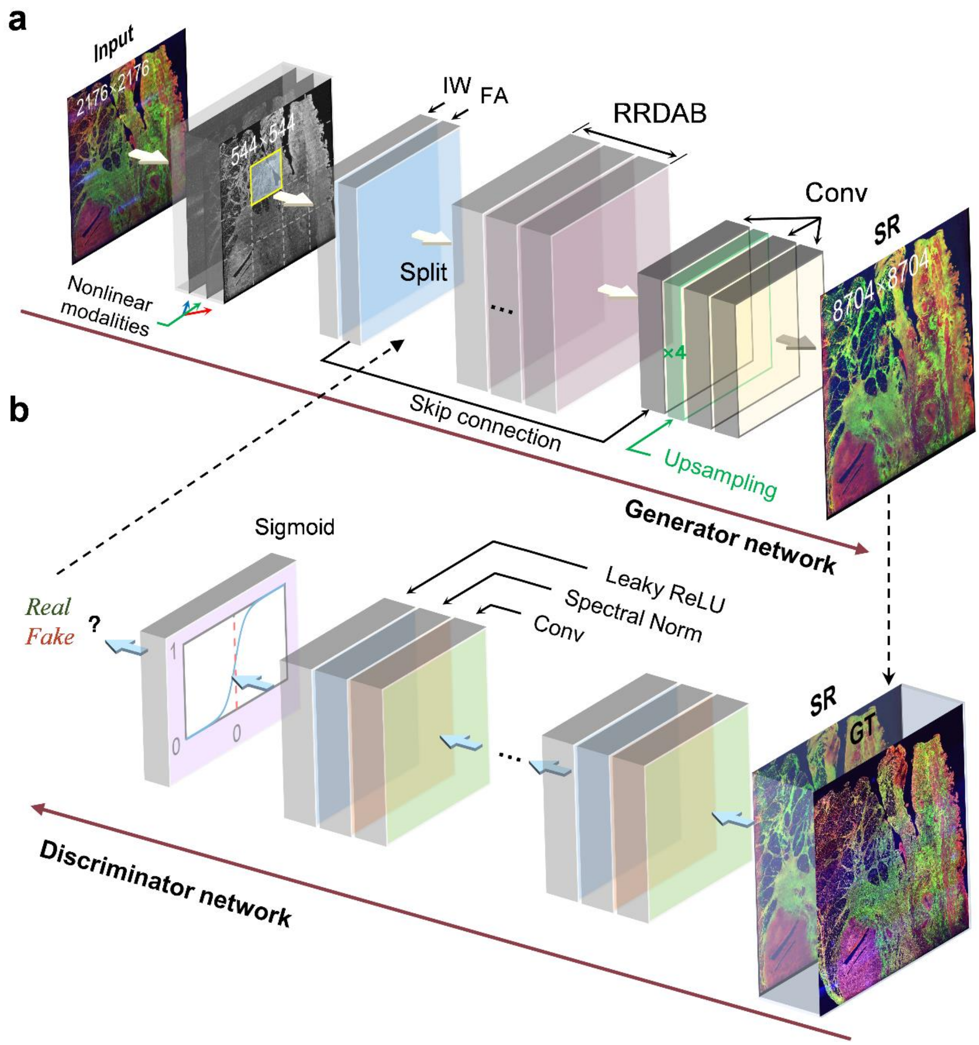

Fig. S1 Generator network and discriminator network frameworks. a The generator network mainly consists of image registration, RRDAB, skip connection, and upsampling and sub-pixel convolution layers. **b** The discriminator network mainly consists of activation function leaky ReLU, spectral normalization, and convolution layers. FA, feature alignment; GT, ground truth; IW, image warping; ReLU, rectified linear unit; RRDAB, residual-in-residual dense attention block; SR, super resolution.

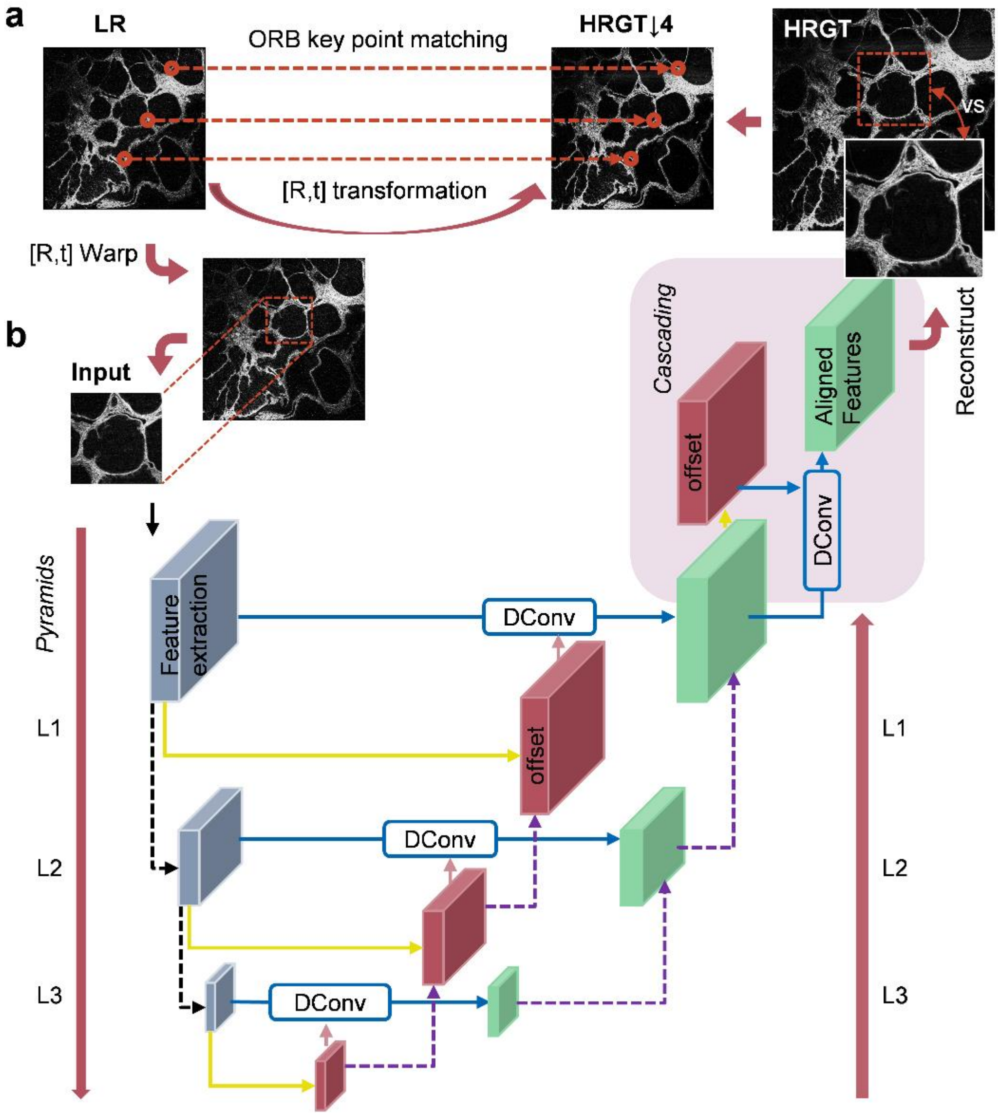

Fig. S2 Image registration framework. a Image warping by the ORB feature extraction method to construct the training dataset. **b** Self-alignment pyramid, cascading, and deformable convolutions (SAPCD) embedded in the generator. DConv, deformable convolution; L, level. LR, low-resolution; HR, high-resolution.

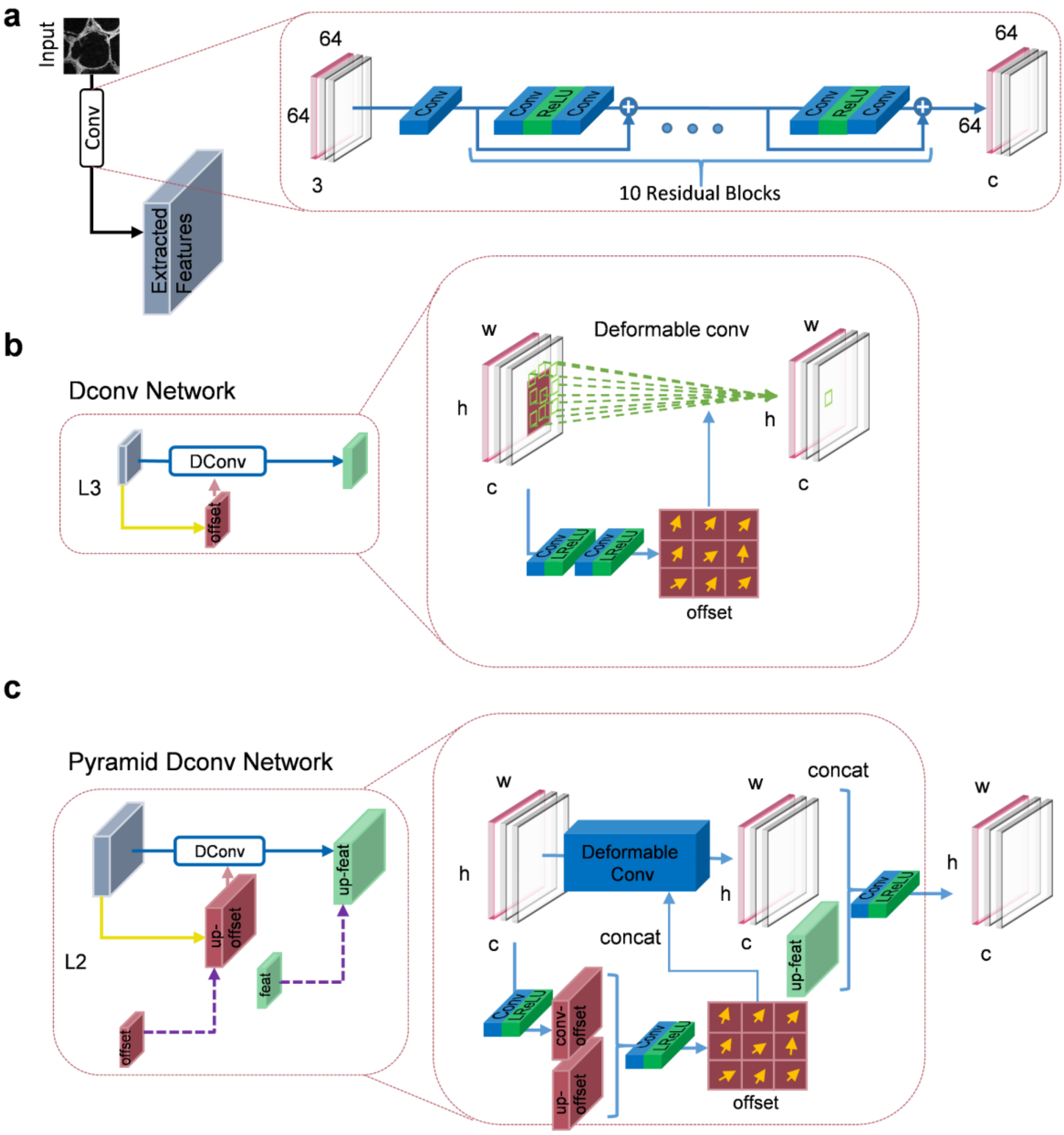

Fig. S3 Detailed network modules in SAPCD. a Feature extraction module with ten residual networks. Deformable convolution network (**b**) and pyramid deformable convolution network (**c**) in L3 and L2 of SAPCD, respectively.

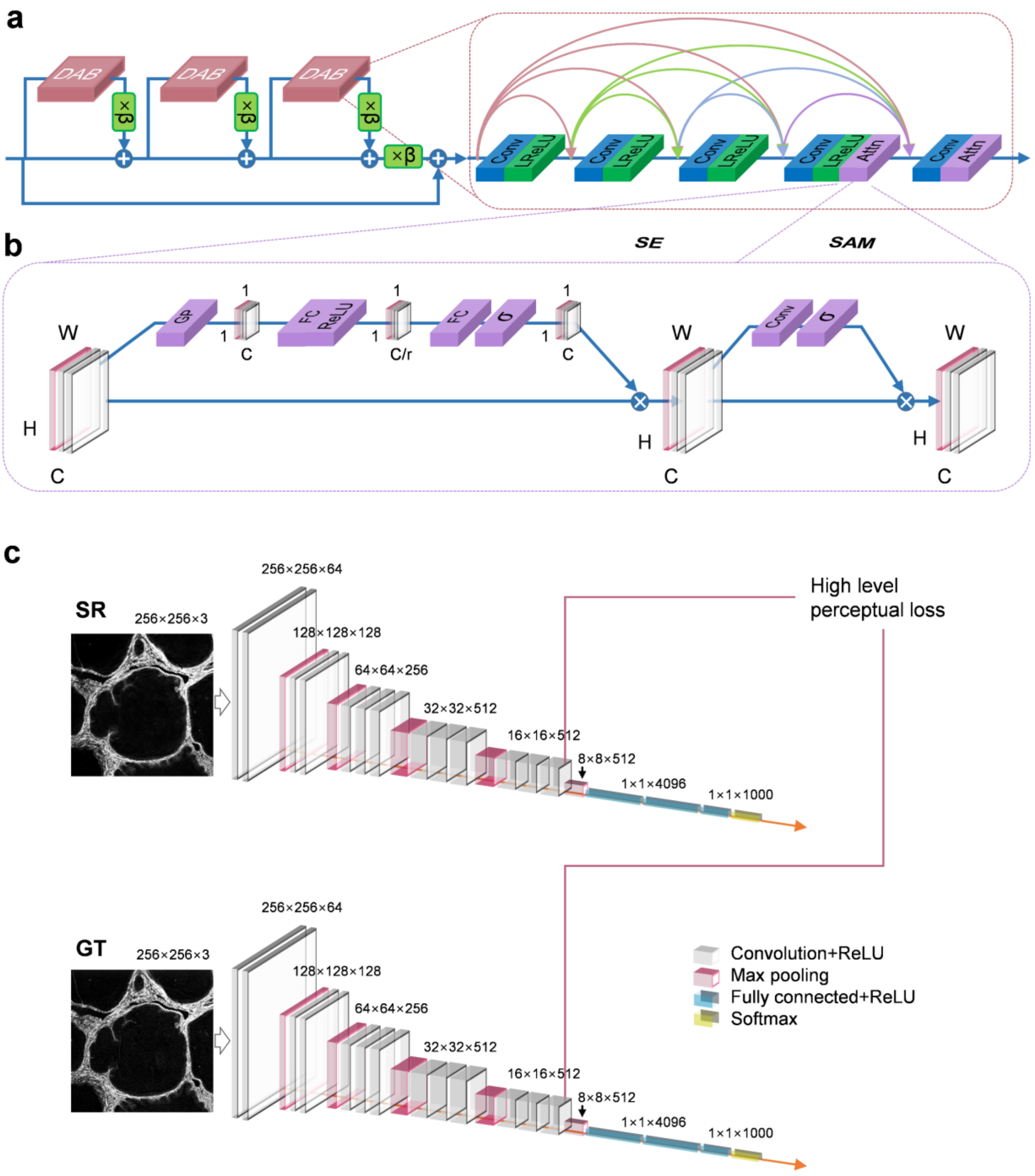

Fig. S4 RRDAB reconstruction and perceptual loss. a Left: cascaded RRDAB modules for image reconstruction. Right, dense connectivity for feature communication. **b** The attention block consists of SE and SAM modules (the SAM module can be substituted with another SE for similar quality improvement). **c** High level perceptual loss used in this work. DAB, dense attention block; Attn, attention; LReLU, leaky ReLU; GP, global pooling; FC, fully connected layer.

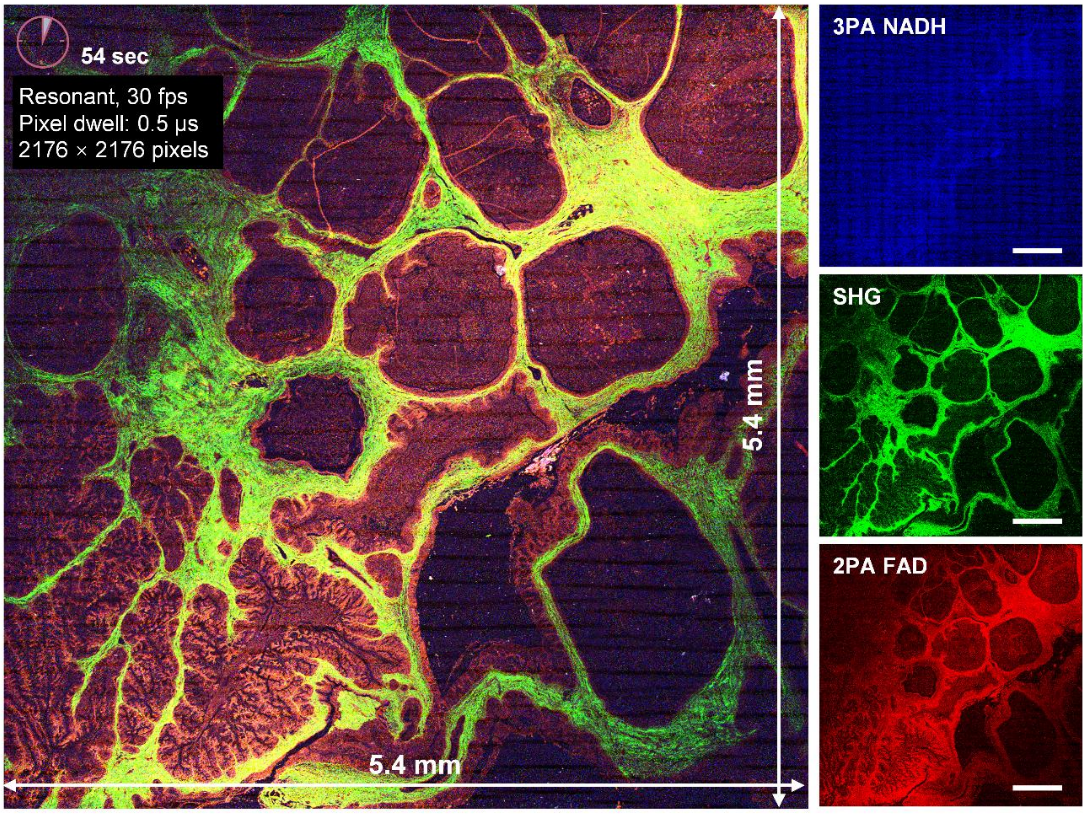

Fig. S5 The input label-free multimodal image of the ovarian borderline carcinoma. Top left shows acquisition time and parameters. The large image on the left is merged by the 3PA NADH, SHG, and 2PA FAD channels shown on the right. Scale bars, 1 mm.

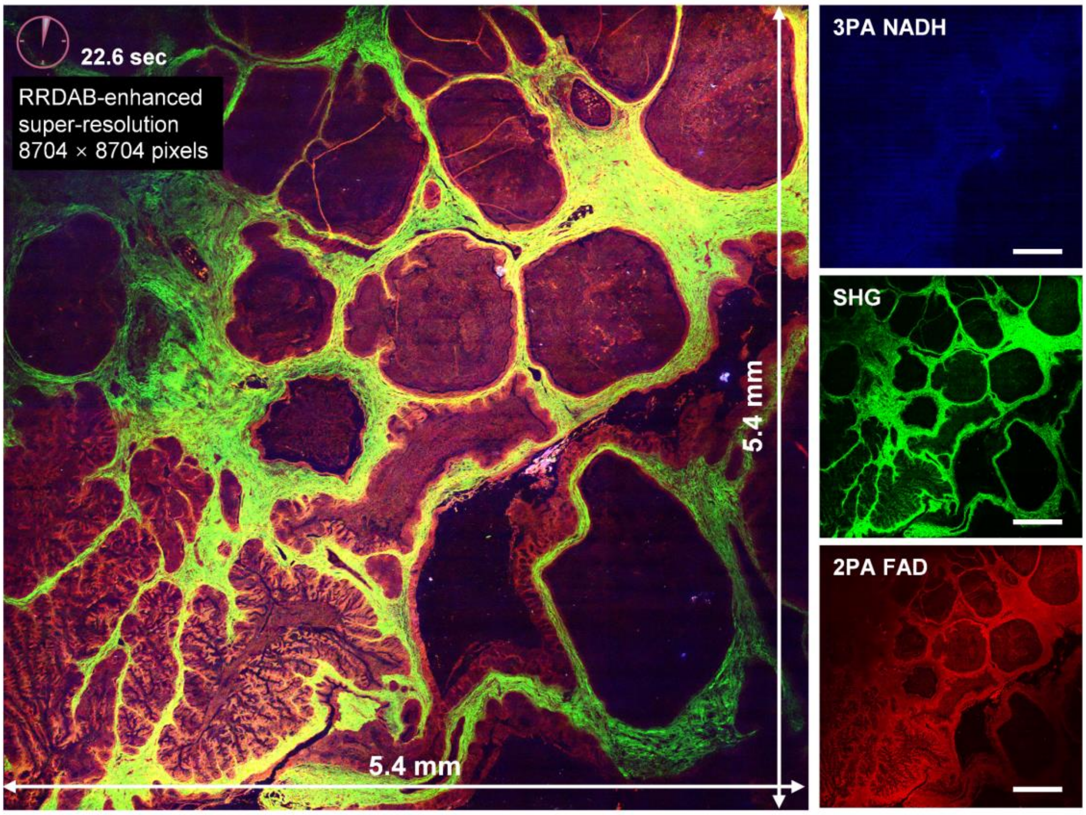

Fig. S6 The reconstruction image of the ovarian borderline carcinoma. Top left shows inference time and upsampling pixel number. The large image on the left is merged by the 3PA NADH, SHG, and 2PA FAD channels shown on the right. Scale bars, 1 mm.

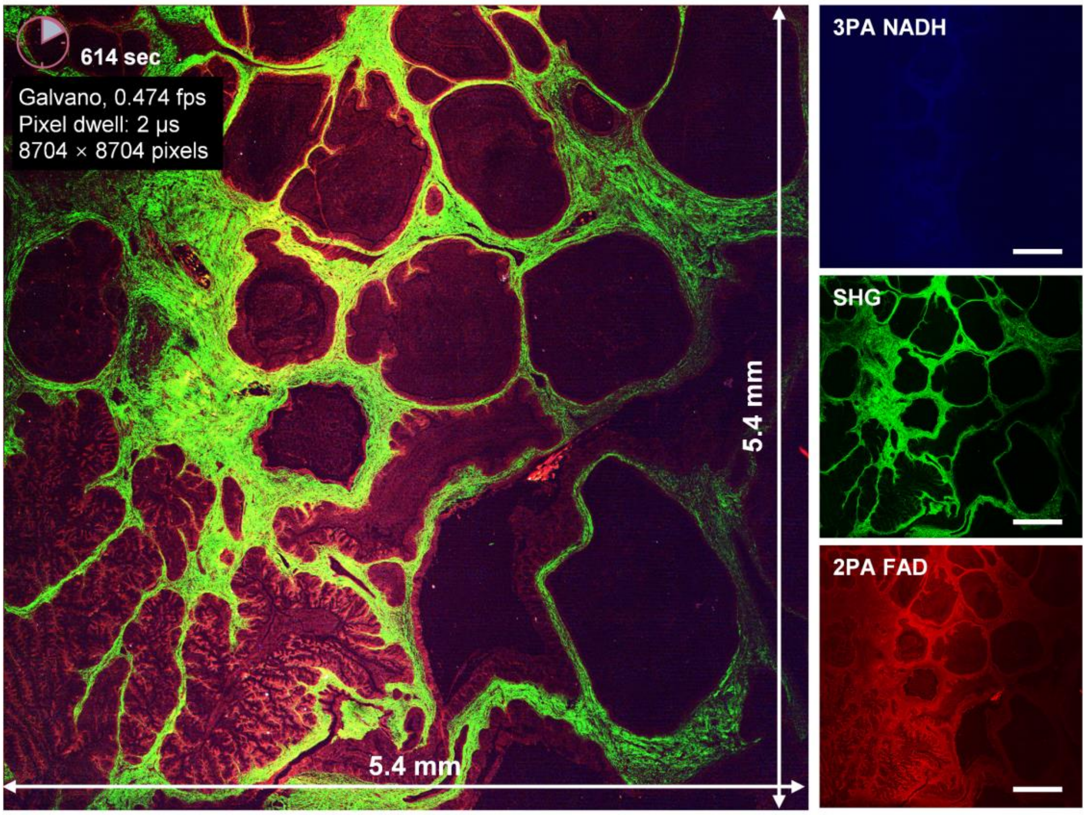

Fig. S7 The GT image of the ovarian borderline carcinoma. Top left shows acquisition time and parameters. The large image on the left is merged by the 3PA NADH, SHG, and 2PA FAD channels shown on the right. Scale bars, 1 mm.

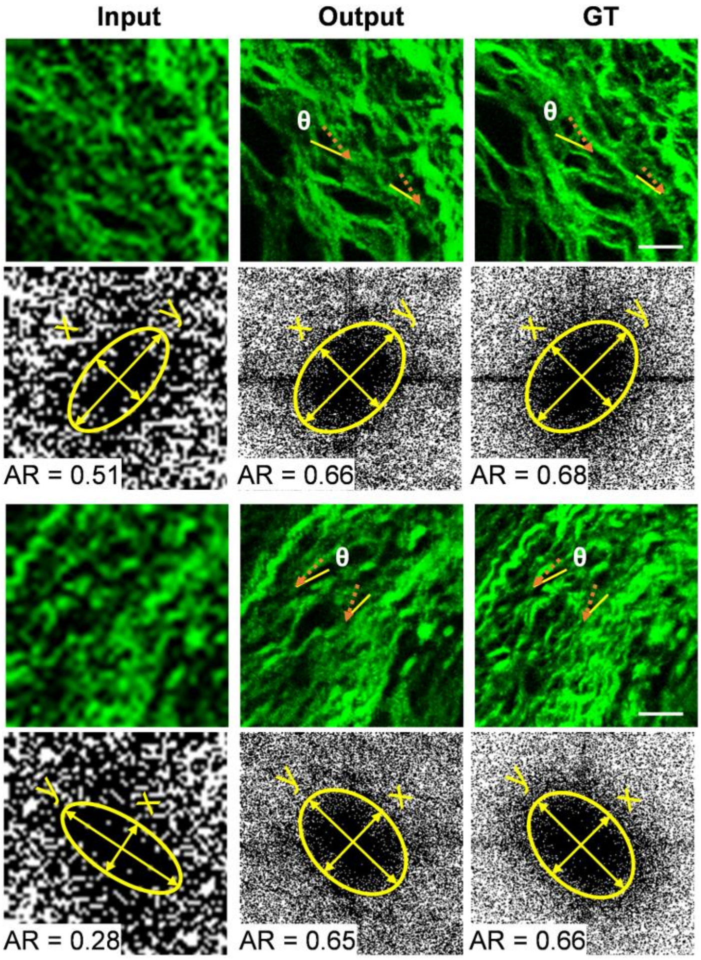

Fig. S8 Quantification of the tumor-associated collagen signatures. The grayscale maps are fast Fourier transform (FFT) result of the corresponding SHG images (green). Orientation (θ) of collagen fiber proliferation (orange dashed arrows) with regard to the epithelium alignment (yellow lines) are distinct in the network output and GT images. The aspect ratio (AR = x/y) are obtained from the fitting ellipse (yellow circles) of the FFT maps. Scale bars, 20 μm.

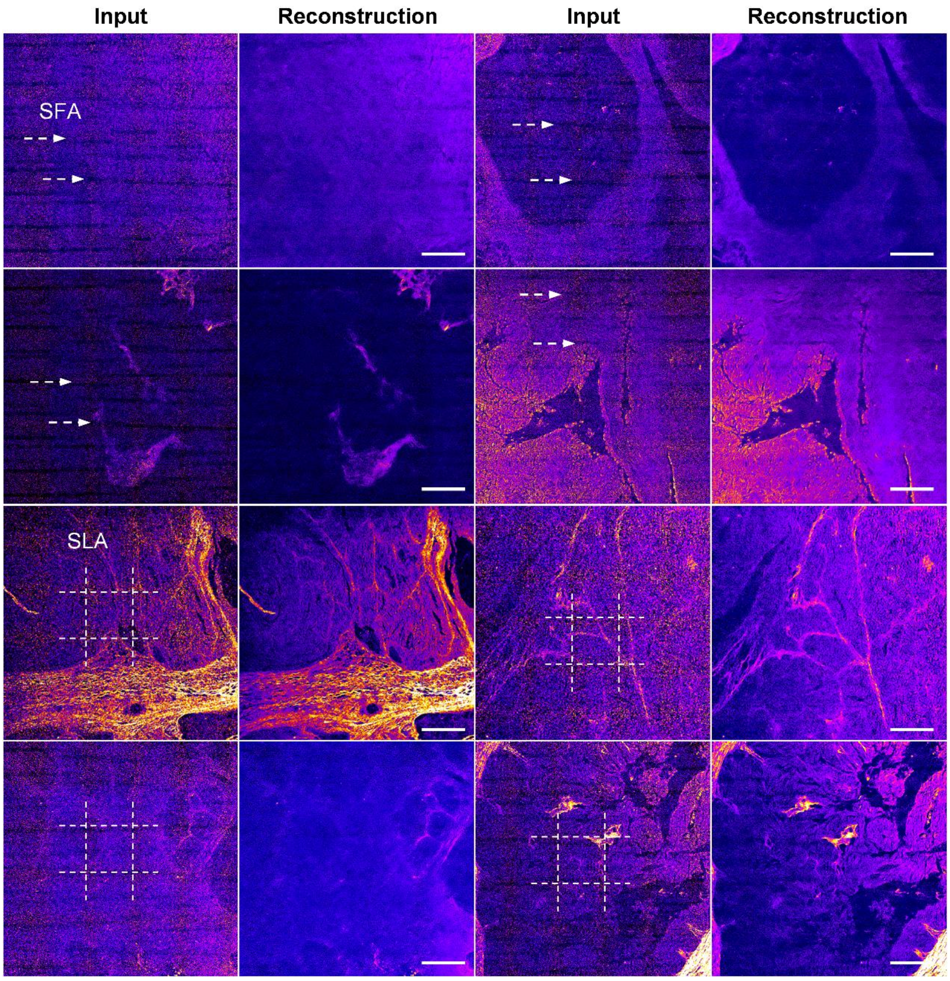

Fig. S9 Scanning fringe artifacts (SFA) and stitching lattice artifacts (SLA) minimization. White dashed arrows indicate the SFA and white dashed lines indicate the SLA. Comparisons between the input and reconstructed images demonstrate a significant decrease in these artifacts. Scale bars, 300 μm.

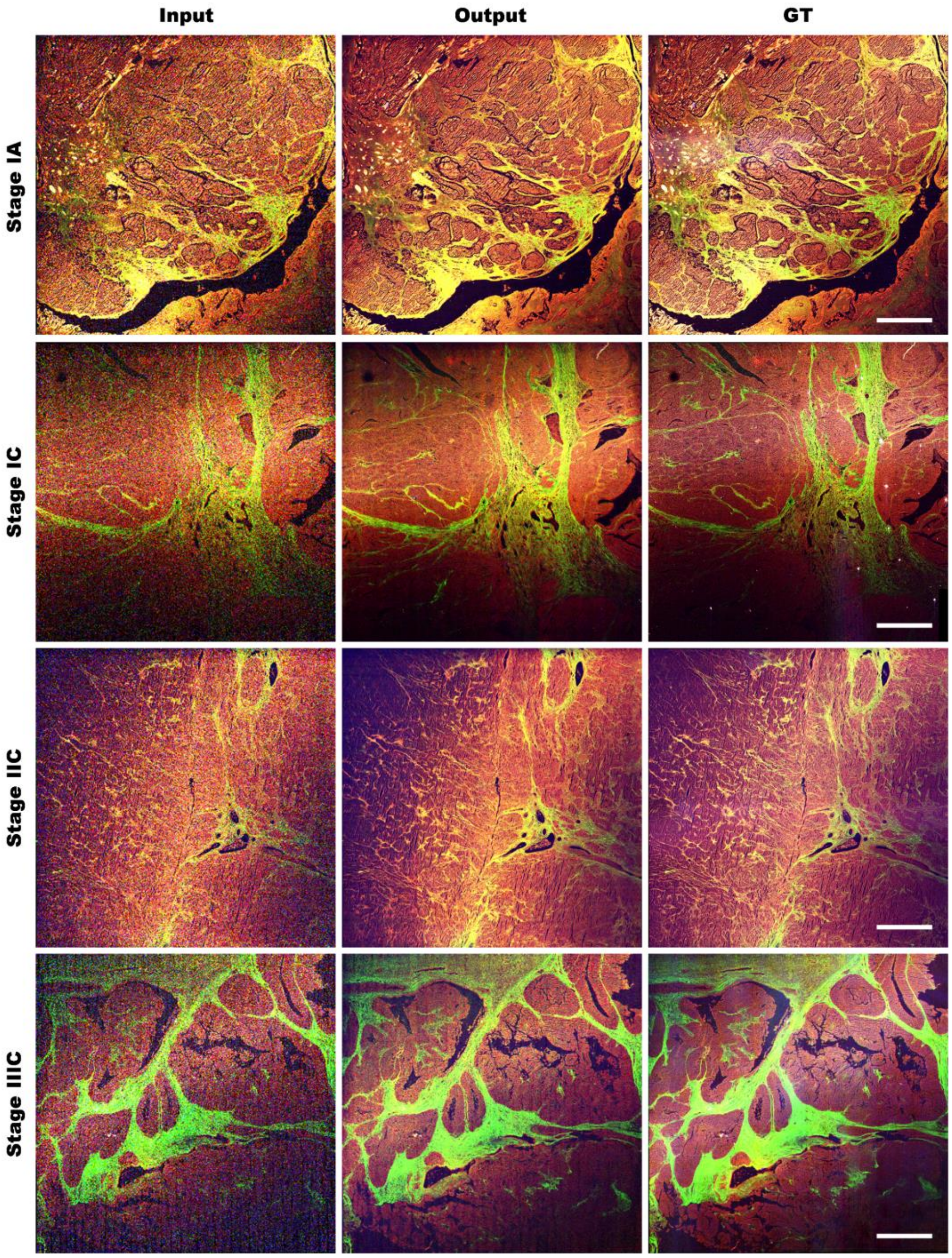

Fig. S10 Label-free multimodal nonlinear images of the ovarian carcinomata at 5.4 × 5.4 mm^2 large field scanning. Pseudocolor presentations of red, green, and blue were merged by 2PA FAD, SHG, and 3PA NADH, respectively. Top to bottom: borderline carcinoma, FIGO Stage IA, IIIC, IV. Left to right: registered input, network output, GT. Scale bar, 1 mm.

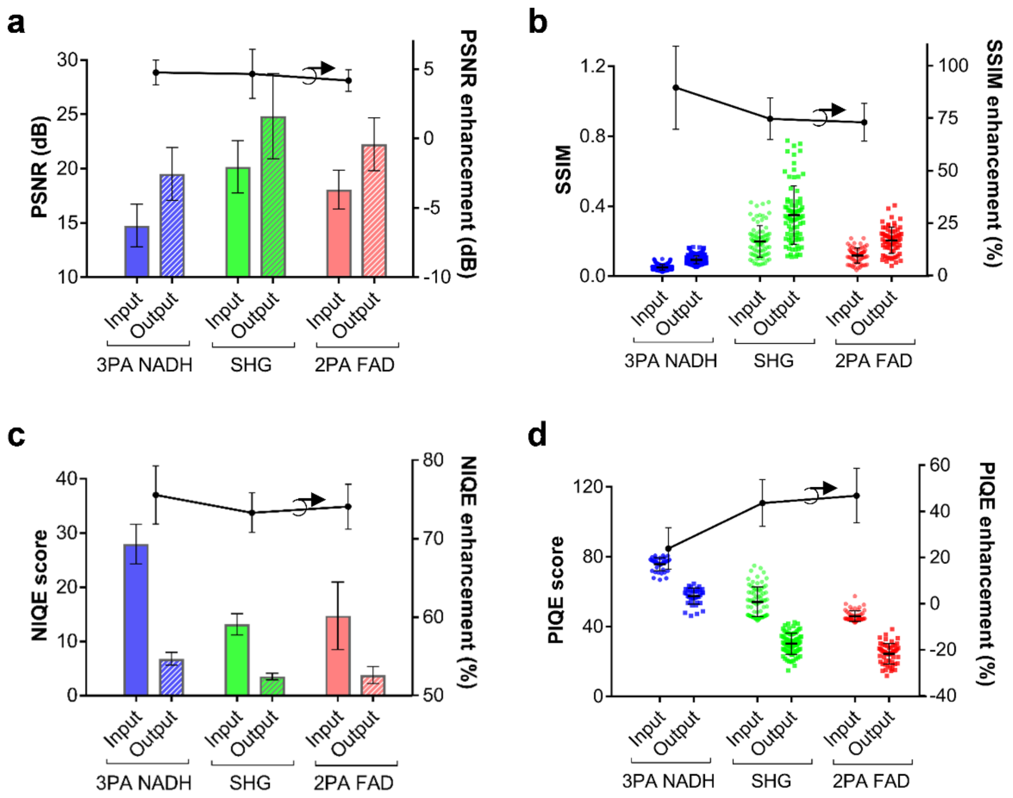

Fig. S11 Statistical image quality improvement by DLAM. Comparison of PSNR (**a**), SSIM (**b**), NIQE (**c**), and PIQE (**d**) between the registered input images (272 × 272 pixels) and network output images (1088 × 1088 pixels) in the 3PA NADH (blue), SHG (green), and 2PA FAD (red) modality. Total n = 439 (PSNR), n = 374 (SSIM), n = 397 (NIQE), and n = 173 (PIQE) paired image tiles with high noise level were selected to demonstrate the image quality improvement. Two-tailed Wilcoxon matched-pairs signed rank test was applied between input and output metrics, $p < 0.0001$ for all paired columns with the same modality in **a**–**d**.

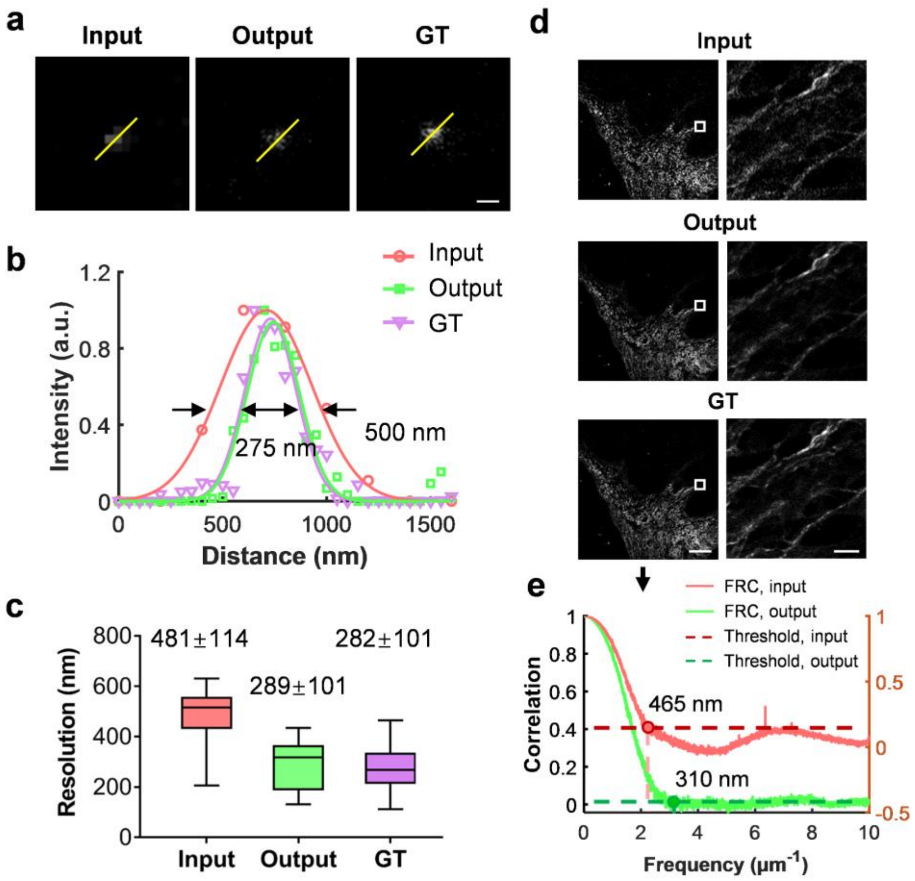

Fig. S12 Evaluation of the resolution improvement. We extracted bright spots (**a**) from the same locations of the network input (quarter downsampled from the GT), network output, and the corresponding GT images. Each one of the cross-sectional profile (yellow lines) of these spots was fit to a Gaussian function (**b**), and the corresponding statistical resolutions are shown in each histogram (**c**). Box plots denote the median, 25th–75th percentiles and minimum and maximum values, n = 14. (**d**) Left column: raw input, network output, and GT SHG images, containing 5×5 FOVs, with different ROIs (white squares) magnified in right column. (**e**) FRC measure on the whole SHG images in (d). Scale bars, 50 nm (a), 100 μm (left column in d), 5 μm (right column in d).

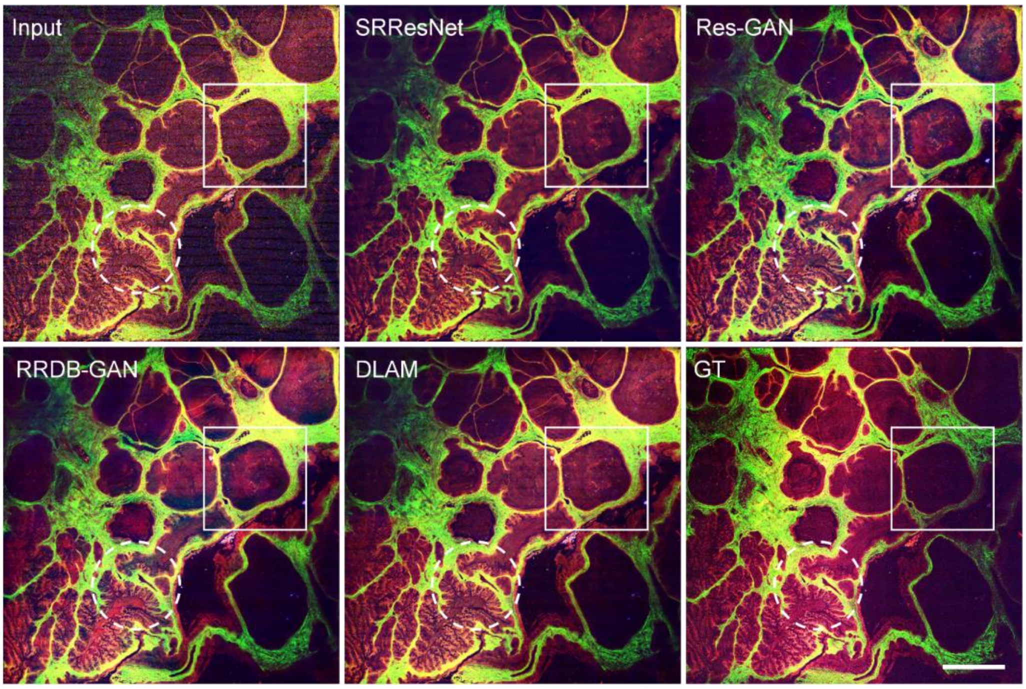

Fig. S13 Comparison of the output results from different networks. White squares indicate the ROIs shown in Fig 5a. White dashed circles indicate another ROI that exhibits obvious reconstruction artifacts produced by these networks except DLAM. Scale bars, 1 mm.

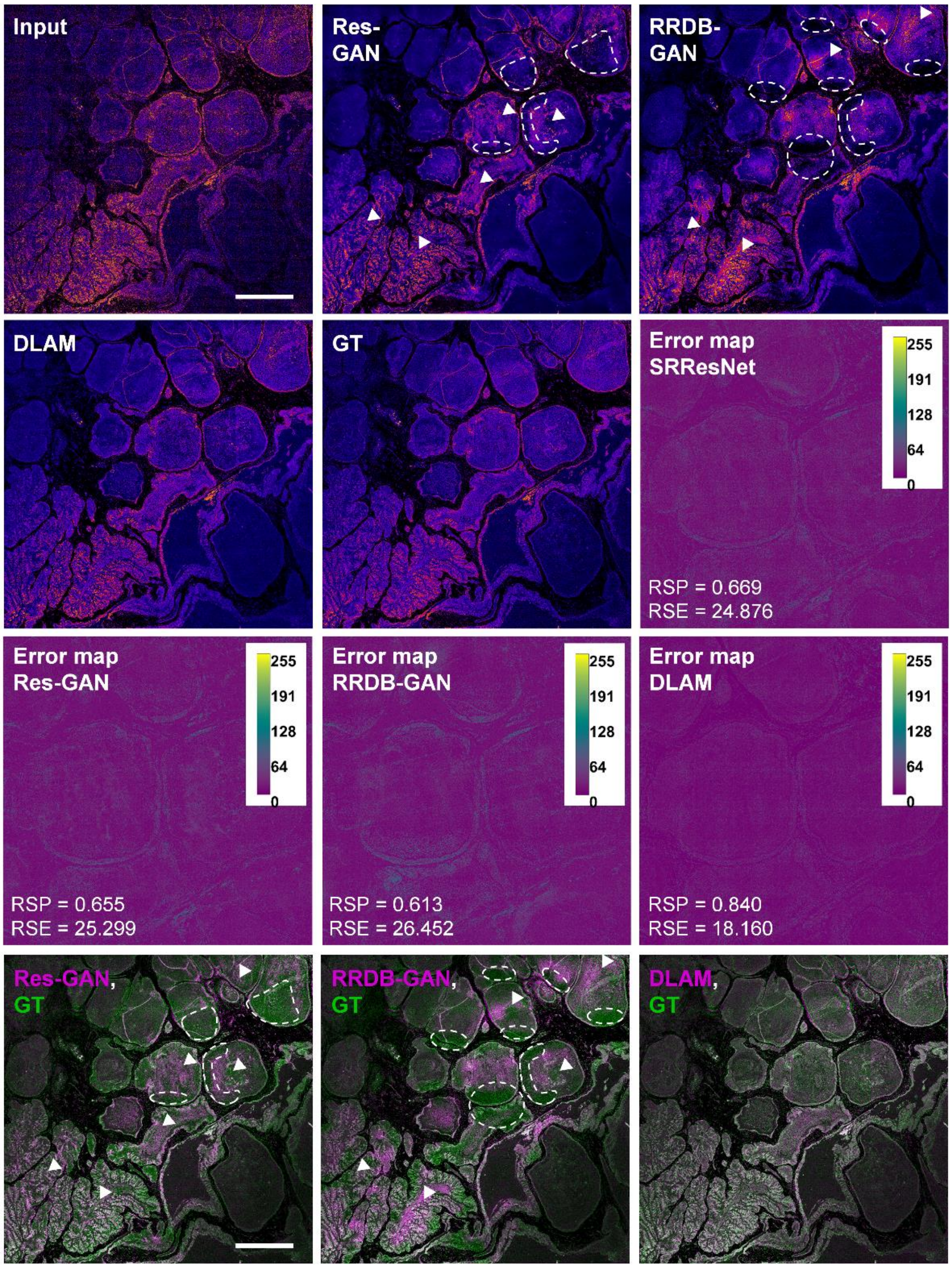

Fig. S14 Quantification of the reconstruction artifacts. First and second columns are image subtractions between 2PA FAD and SHG modality to eliminate overexposure and other instrumental influences. Triangles indicate the hyperplasias and dashed circles indicate the ablation by reconstruction, which are not observed in GT and DLAM. Error maps of the GT and different networks (reference: input) suggest that DLAM has higher RSP scores and less RSE compared to the other networks. Bottom images are merged by the network output image in magenta and the GT image in green, revealing no obvious feature mismatch between DLAM and GT. Scale bars, 1 mm.

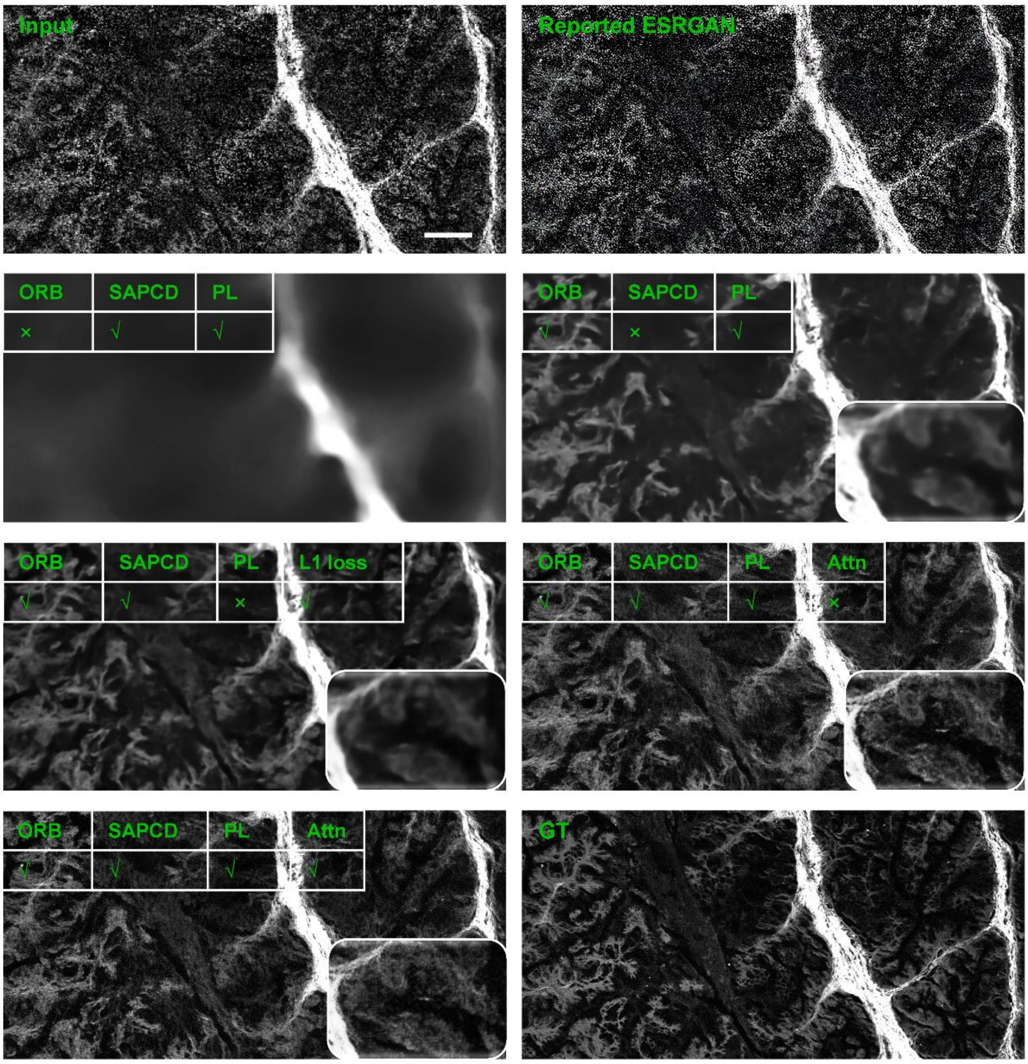

Fig. S15 Ablation study. The symbols in the white boxes indicate the modules which were used (√) or unused (×) in the deep networks. First row: input image (left) and output image by a reported ESRGAN[14] (right). Second row: RRDB-GAN without ORB (left) and without SAPCD (right). Third row: RRDB without perceptual loss (left) and RRDB-GAN with perceptual loss but without attention (right). Final row: DLAM (left) and GT (right). The insets show the 2.6× magnified ROI. PL, perceptual loss. Scale bar, 100 μm.

Table S1 Acquisition/reconstruction time for input, GT, and deep learning.

**Time (s)**	**Single image, single modality**	**Single image, Tri-modality**	**Large image, single modality**	**Large image, Tri-modality**
Captured input (GR scanning)	-	0.033	-	54
Reconstruction (dual-SE)	0.208	0.624	15.04	45.12
Reconstruction (SE-SAM)	0.257	0.771	18.56	55.68
Reconstruction (SE-SAM)*	0.115	0.345	8.32	24.96
**Reconstruction (SE-SAM)****	**-**	**0.312**	**-**	**22.56**
Captured GT (DG scanning)	-	2.1	-	614

* marks computation with RTX 3090. ** marks batch processing (one batch size for one nonlinear modality) using GTX 1080TI GPU. Dual-SE: attention module realized by dual-SE framework cascade; SE-SAM: attention module realized by SE and SAM framework cascade.

Table S2 Image quality metrics on average across the large-field images in Fig. 3.

**Score**	**Full-reference quality metrics**			**No-reference quality metrics**			**Reconstruction artifacts**	
	**MSE**	**PSNR (dB)**	**SSIM**	**BRISQUE**	**NIQE**	**PIQE**	**RSP**	**RSE**
Registered input	0.034	14.78	0.143	43.45	6.10	47.94	-	-
SRResNet	0.021	16.90	0.325	31.72	16.44	70.72	0.740	27.67
ResNet-GAN	0.030	15.64	0.239	29.19	2.43	40.62	0.742	27.58
RRDB-GAN	0.028	15.94	0.247	30.89	2.30	40.41	0.713	28.85
**DLAM (dual-SE)**	**0.023**	**16.67**	**0.205**	**33.94**	**3.01**	**47.27**	**0.880**	**19.64**
**DLAM (SE-SAM)**	**0.024**	**16.47**	**0.210**	**35.12**	**2.70**	**45.88**	**0.880**	**19.59**

Supplementary references

1. Rublee, E., Rabaud, V., Konolige, K. & Bradski, G. ORB: an efficient alternative to SIFT or SURF *in 2011 Ieee International Conference on Computer Vision* 2564-2571 (IEEE, Barcelona; 2011).
2. Lowe, D.G. Distinctive image features from scale-invariant keypoints. *International Journal of Computer Vision* **60**, 91-110 (2004).
3. Song, Z.L. & Zhang, J.P. Remote Sensing Image Registration Based on Retrofitted SURF Algorithm and Trajectories Generated From Lissajous Figures. *Ieee Geoscience and Remote Sensing Letters* **7**, 491-495 (2010).
4. Fischler, M.A. & Bolles, R.C. Random sample consensus: a paradigm for model fitting with applications to image analysis and automated cartography. *Communications of the Acm* **24**, 381-395 (1981).
5. Brown, K.M. & Dennis, J.E. Derivative free analogues of the Levenberg-Marquardt and Gauss algorithms for nonlinear least squares approximation. *Numerische Mathematik* **18**, 289-& (1972).
6. Dai, J.F. et al. in 2017 Ieee International Conference on Computer Vision 764-773 (2017).
7. Bertasius, G., Torresani, L. & Shi, J.B. in Computer Vision - Eccv 2018, Pt Xii, Vol. 11216. (eds. V. Ferrari, M. Hebert, C. Sminchisescu & Y. Weiss) 342-357 (2018).
8. Zhao, Y., Xiong, Y.J. & Lin, D.H. in Advances in Neural Information Processing Systems 31, Vol. 31. (eds. S. Bengio et al.) (2018).
9. Tian, Y.P., Zhang, Y.L., Fu, Y., Xu, C.L. & Ieee in 2020 Ieee/Cvf Conference on Computer Vision and Pattern Recognition 3357-3366 (2020).
10. Wang, X.T. et al. EDVR: Video Restoration with Enhanced Deformable Convolutional Networks *in 2019 Ieee/Cvf Conference on Computer Vision and Pattern Recognition Workshops* 1954-1963 (IEEE, Long Beach; 2019).
11. Zhu, X.Z., Hu, H., Lin, S., Dai, J.F. & Soc, I.C. in 2019 Ieee/Cvf Conference on Computer Vision and Pattern Recognition 9300-9308 (2019).
12. Lai, W.S., Huang, J.B., Ahuja, N., Yang, M.H. & Ieee in 30th Ieee Conference on Computer Vision and Pattern Recognition 5835-5843 (2017).
13. Yang, F.Z. et al. in 2020 Ieee/Cvf Conference on Computer Vision and Pattern Recognition 5790-5799 (2020).
14. Wang, X. et al. ESRGAN: Enhanced Super-Resolution Generative Adversarial Networks *in*

Computer Vision – ECCV 2018 Workshops. (eds. L. Leal-Taixé & S. Roth) 63-79 (Springer International Publishing, Cham; 2019).

15. Jie, H., Li, S., Gang, S. & Albanie, S. Squeeze-and-Excitation Networks. *Ieee T Pattern Anal* **42**, 2011–2023 (2017).

16. Woo, S.H., Park, J., Lee, J.Y. & Kweon, I.S. CBAM: Convolutional Block Attention Module *in Computer Vision - Eccv 2018, Pt Vii*, Vol. 11211. (eds. V. Ferrari, M. Hebert, C. Sminchisescu & Y. Weiss) 3-19 (2018).

17. Dey, N., Zhang, Y.D., Rajinikanth, V., Pugalenthi, R. & Raja, N.S.M. Customized VGG19 Architecture for Pneumonia Detection in Chest X-Rays. *Pattern Recognition Letters* **143**, 67-74 (2021).

18. Ledig, C. et al. Photo-Realistic Single Image Super-Resolution Using a Generative Adversarial Network *in 30th Ieee Conference on Computer Vision and Pattern Recognition* 105-114 (IEEE, Honolulu; 2017).

19. Dong, C., Loy, C.C., He, K. & Tang, X. Image Super-Resolution Using Deep Convolutional Networks. *IEEE Trans Pattern Anal Mach Intell* **38**, 295-307 (2016).

20. Mittal, A., Soundararajan, R. & Bovik, A.C. Making a "Completely Blind" Image Quality Analyzer. *Ieee Signal Processing Letters* **20**, 209-212 (2013).

21. Barretto, R.P.J., Messerschmidt, B. & Schnitzer, M.J. In vivo fluorescence imaging with high-resolution microlenses. *Nature Methods* **6**, 511-U561 (2009).

22. Chen, J.J. et al. Three-dimensional residual channel attention networks denoise and sharpen fluorescence microscopy image volumes. *Nature Methods* **18**, 678–687 (2021).

23. Wang, H. et al. Deep learning enables cross-modality super-resolution in fluorescence microscopy. *Nat Methods* **16**, 103-110 (2019).

24. Qiao, C. et al. Evaluation and development of deep neural networks for image super-resolution in optical microscopy. *Nature Methods* **18**, 194-202 (2021).

25. Koho, S. et al. Fourier ring correlation simplifies image restoration in fluorescence microscopy. *Nature Communications* **10**, 3103 (2019).
